# Selective Biomass Valorization via Cascade Photooxidation and Photothermal Hydride Shift

**DOI:** 10.1021/jacs.6c01516

**Published:** 2026-03-27

**Authors:** Yingchuan Zhang, Fupeng Zhang, Ruotong Yang, Guangri Jia, Zhuang Ma, Nengchao Luo, Zhengxiao Guo

**Affiliations:** † Department of Chemistry, 25809 The University of Hong Kong, Pokfulam 999077, Hong Kong, China; ‡ State Key Laboratory of Catalysis, Dalian National Laboratory for Clean Energy, Dalian Institute of Chemical Physics, Chinese Academy of Sciences, Dalian 116023, China

## Abstract

Photocatalytic upgrading of biomass-derived carbohydrates and glycerol into lactic acid (LA) offers a sustainable route to biodegradable plastics and avoids the high temperature/pressure and stoichiometric bases required by traditional thermocatalysis. However, aqueous photoreforming at neutral pH suffers from poor selectivity because the key intermediate, pyruvaldehyde (PYA), undergoes multiple redox reactions in parallel with intramolecular disproportionation to produce LA. By introduction of effective Lewis-acid sites (unsaturated Ti^4+^), a Cannizzaro-type reaction is enabled with cascade photooxidation and 1,2-hydride shift for highly selective production of LA over proton-coupled electron transfer (PCET) intermediates and ^•^OH-overoxidized products. Upon irradiation, the Lewis-acid sites modulate photooxidation and intermediate binding, whereas the plasmonic Au nanoparticles induce localized heat to promote the rate-limiting 1,2-hydride shift, thus preventing overoxidation. The overall cascade leads to >90% LA selectivity, a 3.4-fold increase from solely photocatalytic processes, and an unprecedented productivity of 130.8 mmol g^–1^ h^–1^ under ambient conditions. This work highlights the potential of multifunctional catalysts to steer complex and parallel reaction networks toward efficient solar biorefineries.

## Graphical abstract

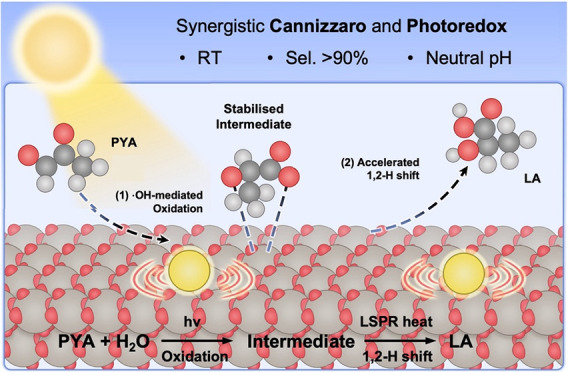

## Introduction

To mitigate the demand on nonrenewable petrochemicals, it is increasingly important to develop biobased plastics, e.g., polyethylene furanoate (PEF), polybutylene succinate (PBS), and polylactic acid (PLA).^1,2^ Among those, PLA possesses the highest production volume of 2.59 million tons in 2025 with a compounded annual growth rate of 20% due to the robust strength and biodegradable properties in closed-loop recycling sectors.^3,4^ Traditional technologies for the production of lactic acid (LA) include the fermentation of lignocellulosic biomass by filamentous fungi and the liquid-phase reforming of carbohydrates and glycerol, which demand the input of high-concentration bases and/or Lewis-acid catalysts operated at high temperatures (>140 °C).^5–7^ In contrast, photocatalytic reforming emerges as an alternative approach for biomass valorization, such as the oxidation of cellulose to formic acid and the reduction of 5-hydroxymethylfurfural (HMF) to 2,5-bis­(hydroxymethyl)­furan (BHMF), which is operated under ambient conditions without external energy-intensive infrastructure.^8,9^ Photocatalysis can upgrade glucose into LA by cascade isomerization, retro-aldol condensation, dehydration, and intramolecular disproportionation at 25–50 °C with strong bases (Scheme 1a and Table S1).^10,11^ Glycerol, a biodiesel byproduct, can also be upgraded to LA via sequential oxidation, dehydration, and intramolecular disproportionation over various photocatalysts (Scheme 1b).^12,13^

**Scheme 1:**
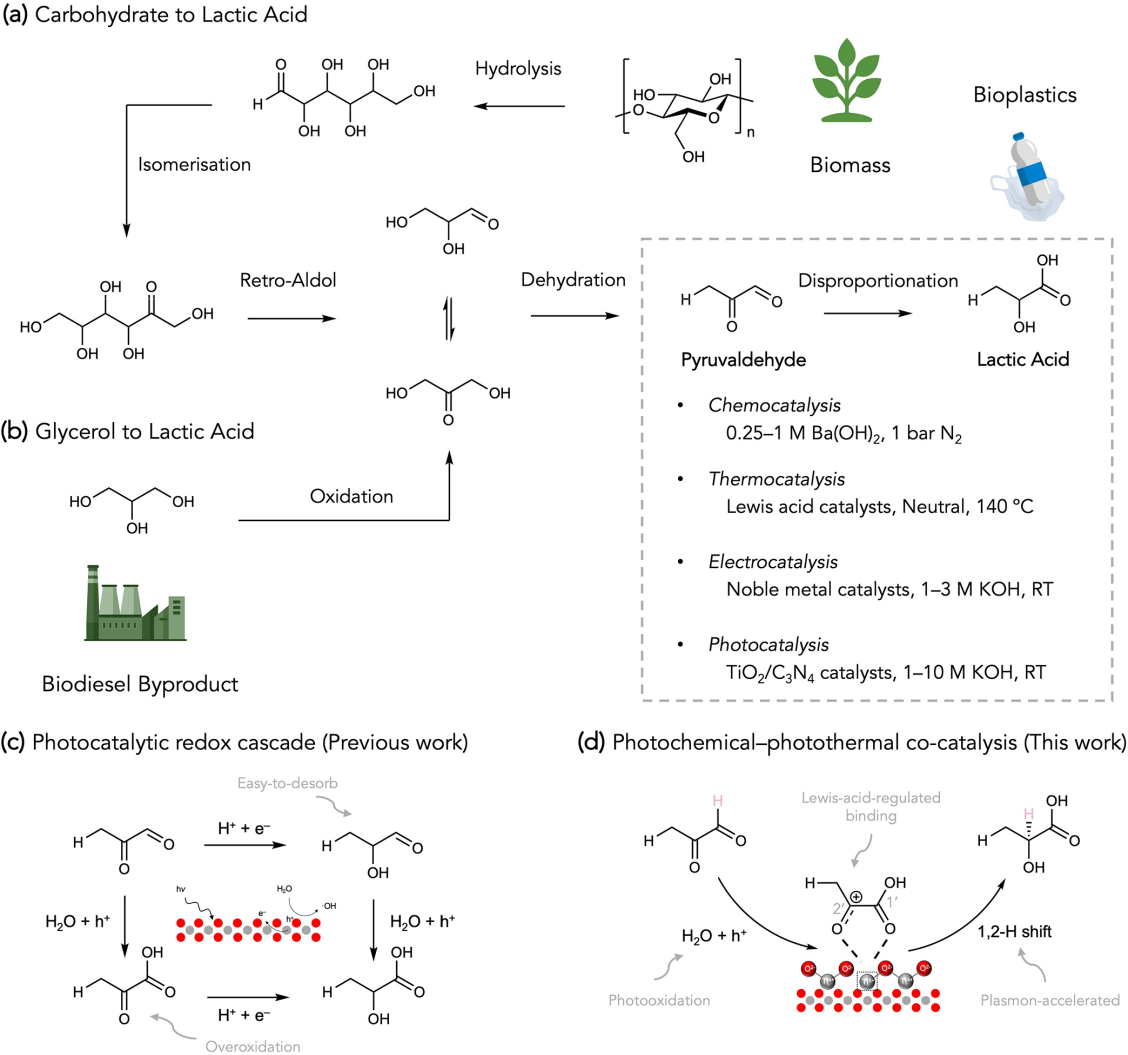
(a, b) Schematic of Carbohydrate and Glycerol to Lactic Acid and Different Catalytic Approaches. (c, d) Illustrations of Photocatalytic Cascaded Disproportionation and Photothermal Cannizzaro-Type Reaction for the PYA-to-LA Conversion

Traditional thermochemical approaches utilize high-concentration alkali salts, e.g., Ba­(OH)_2_ and KOH, as both the catalyst and the reactant to ultimately enable ∼95% LA yields.^14^ In comparison, photocatalytic approaches only achieve a maximal yield of 86%, even in a 10 M KOH solution with glucose as the feedstock.^10^ The low product selectivity of LA is more evident under neutral conditions, where multiple C_3_ oxygenates are reported as side-products.^15,16^ Regardless of feedstocks (glucose, fructose, or glycerol) and catalysts for the photocatalytic production of LA, pyruvaldehyde (PYA) is the key intermediate generated from the dehydration of unstable glyceraldehyde and hydroxyacetone (Scheme 1a,b).^17^ With two aldehyde groups, PYA can undergo asymmetric redox transformations, leading to multiple redox products. Moreover, the reforming of PYA into LA involves complex intramolecular disproportionation via unidentified intermediates and unclear pathways.^18^ Previous reports suggest a thermochemical pathway for PYA disproportionation to LA via *OH attack and 1,2-hydride shift over strong Lewis-acid catalysts.^19,20^ However, photochemical disproportionation has been less studied. The only example is a temperature-tunable process for reforming tertiary alcohols to alkanes and ketones via reductive hydrodeoxygenation and oxidation, respectively.^21,22^ Moreover, hydride chemistry is increasingly important with superior activities in CO_2_ hydrogenation and NADH regeneration.^23,24^ However, the sluggish kinetics of hydride transfer renders it as the rate-determining step, and therefore external energy inputs such as high temperature or electrochemical polarization are needed to promote the overall reaction.

Here, we combine thermal Cannizzaro-type and photoredox steps to enable highly selective disproportionation of PYA to LA (Scheme 1c). Competitive entries and in situ spectroscopy reveal multiple reactive species and cascaded functional-group transformations in thermo-/photo-reforming of PYA over rutile TiO_2_ as a model catalyst. Reaction intermediates and kinetics are identified and associated with Lewis acidity, thermal effects, and photoredox species to explain the competition between Cannizzaro and photoredox reactions under different conditions. Theoretical calculations identify distinct rate-determining steps (RDSs), i.e., 1,2-hydride shift for Cannizzaro reaction and proton-couple electron transfer (PCET) for photoredox pathways, featuring different reaction kinetics and intermediate energetics. Based on the finding, we load photothermally active metals and introduce oxygen vacancies into TiO_2_ to enhance the Lewis acidity. The resulting Ag/TiO_2_–V_O_ and Au/TiO_2_–V_O_ promote cascaded photooxidation and a photothermal 1,2-hydride shift to ultimately deliver 95% LA selectivity under ambient conditions.

## Results and Discussion

In aqueous photoreforming, the two carbonyl groups in PYA, an aldehyde (−CHO) and a ketone (−C=O), can undergo asymmetric redox transformations to produce C_3_ oxygenates, including hydroxyacetone, lactaldehyde, LA, and pyruvic acid (Figures 1a and S1). We initially aimed to develop Pd/Pt-doped TiO_2_ for the coproduction of LA and H_2_ from neutral carbohydrate solutions by leveraging spatially separated photogenerated species and effective hydrogen evolution reaction (HER) sites. However, both 1 wt % Pd/TiO_2_ and 1 wt % Pt/TiO_2_ exhibit LA selectivity of <6% compared to pristine TiO_2_ (∼20%, Figure 1b). This inspires us to reconsider the role of photogenerated electrons (e^–^) and the mechanistic nature of the PYA reforming. Previous reports have identified the glycerol-to-LA conversion as an oxidation process involving only photogenerated holes (h^+^) and reactive oxygen species (ROS), e.g., superoxide (^•^O_2_^–^) and hydroxide (^•^OH) radicals, while e^–^ are transferred to noble-metal sites for HER (H^+^ + e^–^ → 1/2 H_2_).^10,12^ However, the above entries suggest the simultaneous involvement of oxidizing and reducing species, which is aligned with the characteristics of a disproportionation reaction. Compared to direct irradiation of the PYA solution that mainly affords an oxidation product (pyruvic acid), photocatalysis over pristine TiO_2_ generates reduction products (lactaldehyde and hydroxyacetone) with >30% selectivity and a disproportionation product (LA) with only ∼20% selectivity. C_1–2_ acids in addition to pyruvic acid are also detected, suggesting the rapid overoxidation on the unmodified TiO_2_ surface.

**Figure 1:**
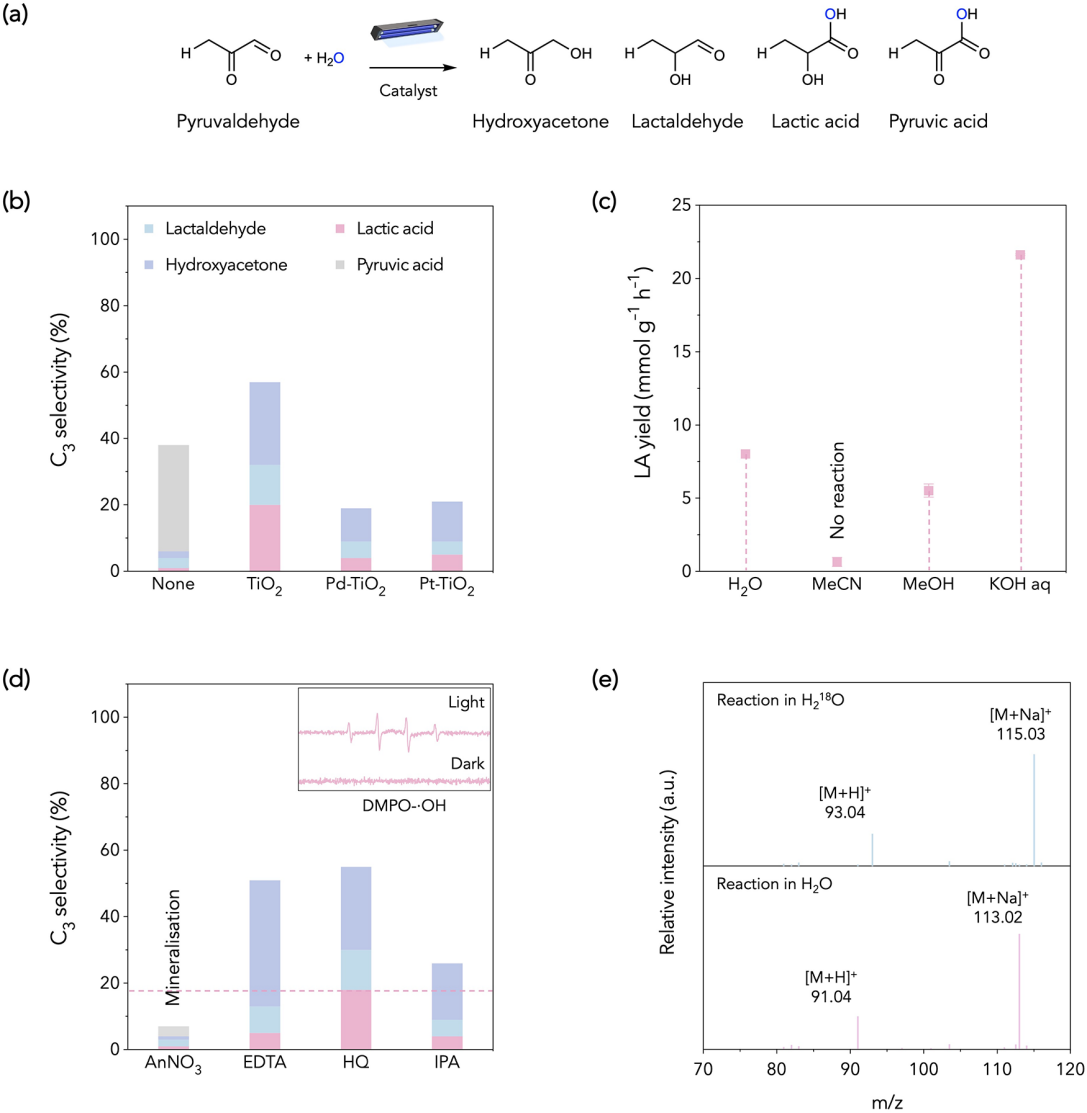
Catalytic performance and reactive species identification in PYA photoreforming. (a) Schematic of the proposed reaction network for photocatalytic reforming of PYA to C_3_ oxygenates, including hydroxyacetone, lactaldehyde, LA, and pyruvic acid. (b) C_3_ product selectivity over catalysts based on commercial rutile TiO_2_ (pristine TiO_2_, 1 wt % Pd/TiO_2_, 1 wt % Pt/TiO_2_). Reaction conditions: 5 mg catalyst, 100 μL PYA solution, 5 mL water, Ar atmosphere, 3 h, RT, UV irradiation. (c) LA production rates in different solvents (H_2_O, MeOH, MeCN) under otherwise identical conditions. (d) EPR spectra showing the DMPO–OH quartet (3460–3560 G) and scavenger test: AgNO_3_ (e^–^ scavenger), EDTA (h^+^ scavenger), hydroquinone (^•^O_2_^–^ scavenger), and isopropanol (^•^OH scavenger), with corresponding effects on C_3_ selectivity. (e) MS spectra of LA-related peaks from isotopic labeling.

The transformations of functional groups were also confirmed by in situ attenuated total reflectance Fourier transform infrared (ATR-FTIR) spectroscopy. As shown in Figure S2, the intensities of C=O stretches at 1707 and 1625 cm^–1^ decrease within 20 min of irradiation in both catalyst and catalyst-free entries, indicating the high reactivity of carbonyl (especially −CHO) groups in aqueous photoreforming. The catalyst-free entry shows remarkably increased intensities of the O–H bend at 1420 cm^–1^ and C–O stretch at 1320 cm^–1^ that are related to nascent −COOH groups, suggesting the formation of pyruvic acid (Figure S2a). In comparison, over TiO_2_, the C=O bands at 1707 and 1625 cm^–1^ remain relatively strong, while the carboxylic O–H bend increases only slightly but with a significant increase around ∼1100 cm^–1^ representing the alcoholic O–H bend (Figure S2b). This indicates that the reduction products are kinetically more favorable than the oxidation product.

To elucidate the photochemical-driven forces, the PYA reforming process was performed over TiO_2_ in varied solvents (Figure 1c,d). In aprotic MeCN, the conversion was completely suppressed, suggesting the necessity of a protic environment for radical generation and proton-coupled steps. MeOH, as an effective hole scavenger, also delivers a lower LA production (5.5 mmol g^–1^ h^–1^) than that in H_2_O (8.0 mmol g^–1^ h^–1^, Figure 1c), indicating the engagement of both electrons and holes in the disproportionation process. Since TiO_2_ can effectively promote one-electron water oxidation (H_2_O + h^+^ → ·OH + H^+^, + 2.73 V_NHE_), we envision that ^•^OH radicals and electrons codrive the PYA disproportionation (Figure S3).^25^ This is further suggested by the 2 M KOH entry, where higher LA selectivity is achieved due to enriched OH^–^ species that can facilitate ^•^OH formation. The conclusion is also supported by previous reports of alkali-assisted photoreforming of glycerol.^11,15^

We further confirmed the presence of ^•^OH trapped by 5,5-dimethyl-1-pyrroline N-oxide (DMPO) in the electron paramagnetic resonance (EPR) spectrum (Figure 1c), and evaluated the PYA reforming process with various radical scavengers (Figure 1d). AgNO_3_, as an electron scavenger, delivers a low LA selectivity of <5% with observed overoxidized products. Adding EDTA as a hole scavenger produces hydroxyacetone instead of LA selectivity via kinetically favored reduction. Hydroquinone, a ^•^O_2_^–^ scavenger, shows a negligible effect on PYA-to-LA conversion, indicating that ^•^O_2_^–^ is not a key oxidant.^26^ In contrast, isopropanol (a ^•^OH scavenger) remarkably decreases LA selectivity to ∼4% but with less impacted production of hydroxyacetone and lactaldehyde (∼22%), suggesting that the ^•^OH-mediated oxidation step is parallel to electron-driven reduction.

To further confirm that PYA is oxidized by water-derived ^•^OH, we performed isotopic labeling in H_2_^18^O. Liquid chromatography–mass spectroscopy (LC-MS) of product mixtures exhibits two series of LA signals, [M + H]^+^ and [M + Na]^+^, each shifted by +2 *m*/*z* relative to H_2_^16^O (Figure 1e). Collectively, these results indicate that both e^–^ and ^•^OH directly participate in the PYA-to-LA conversion via parallel steps, which ultimately limit LA selectivity to <20% in TiO_2_-catalyzed processes at room temperature.

Based on previous reports where ZrO_2_/TiO_2_ composites delivered high LA selectivity via Lewis-acid-catalyzed Cannizzaro-type disproportionation at elevated temperatures (>140 °C), we examined whether a similar strategy works under photocatalytic conditions.^20^ To distinguish thermal pathways from photoredox chemistry on TiO_2_, we conducted temperature-dependent in situ diffuse reflectance infrared Fourier transformations spectroscopy (DRIFTS).^21,22^ As shown in Figure 2a, within 120 min of irradiation at RT, the aldehydic C=O band of PYA at 1710 cm^–1^ diminishes while a nascent and lower-frequency C=O band appears near ∼1660 cm^–1^, which is consistent with the formation of reduced intermediates mentioned above. Meanwhile, the LA-related carboxylic O–H bending band at ∼1435 cm^–1^ shows only a modest increase, indicating the competitive production of multiple C_3_ products. By contrast, heating from 25 to 80 °C in the dark drives the selective LA formation with rare reduction products. The aldehydic C=O band at ∼1710 cm^–1^ vanishes, while a broad carboxylic O–H stretch emerges in the 2500–3300 cm^–1^ region (observed within 2850–2950 cm^–1^ and overlapping aliphatic C–H stretches) together with an increased O–H bend at 1435 cm^–1^ (Figure 2b). Broad features assigned to C–O stretches around 1100–1150 cm^–1^ are present at 25–50 °C but diminish at higher temperatures, suggesting the transient formation of OH–bonded intermediates as a characteristic of the Cannizzaro reaction. Overall, these observations support that thermal reforming of PYA over TiO_2_ can prevent the formation of reduction intermediates compared to photochemical processes and deliver higher LA selectivity at elevated temperatures.

**Figure 2:**
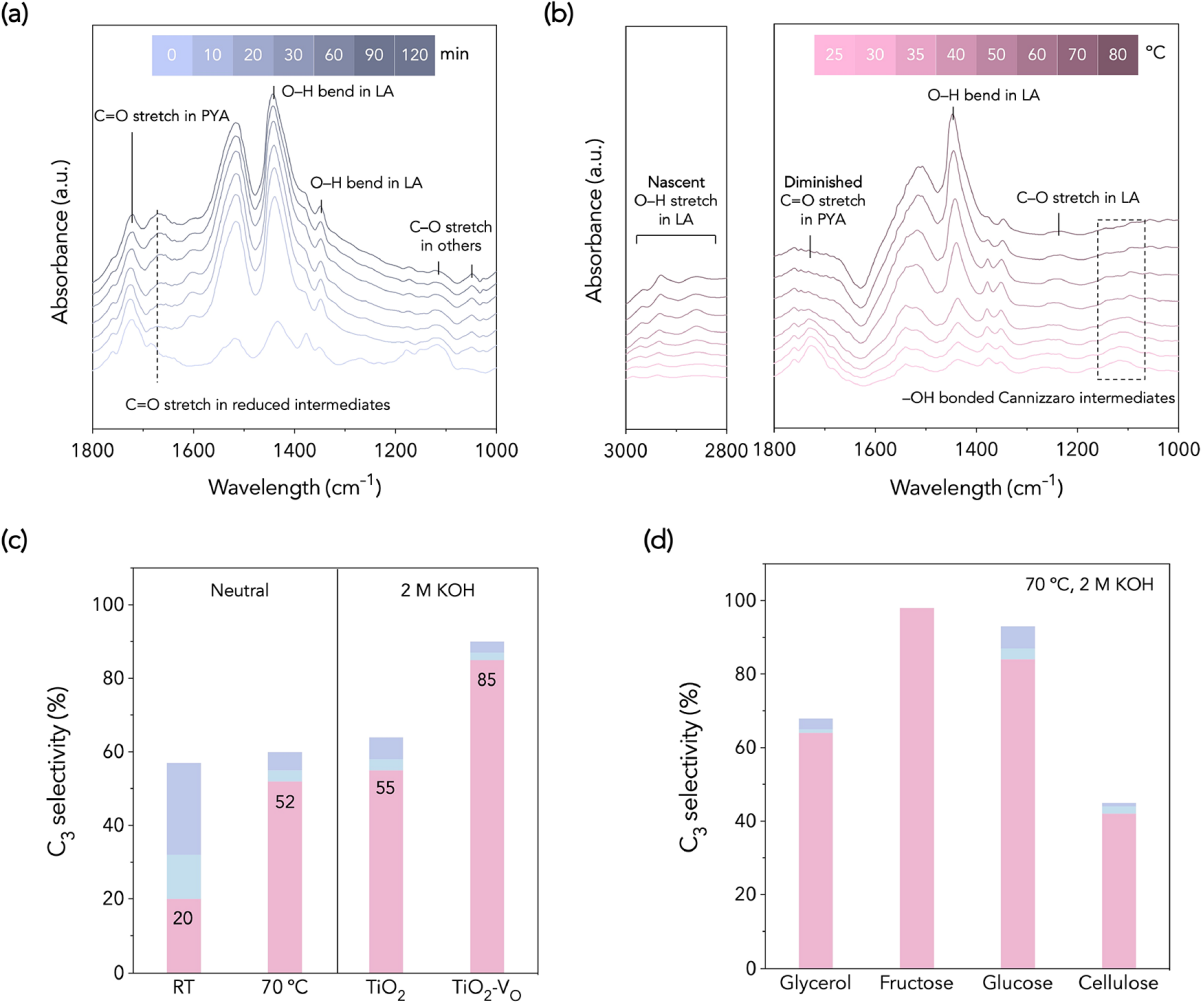
Spectroscopic studies and performance evaluation of Cannizzaro and photoredox reactions. (a) In situ DRIFTS of PYA reforming under irradiation at RT in 120 min and (b) under irradiation with external heating from 25 to 80 °C, both operated in Ar with TiO_2_ rutile as a catalyst. (c) Effects of temperature and Lewis acidity on PYA reforming processes. (d) Substrate expanding under optimized conditions (5 mg catalyst, 100 mg substrate, 5 mL 2 M KOH solution, Ar atmosphere, 3 h, 70 °C, 365 nm light irradiation).

To explore the synergy between thermal and photoredox pathways, we carried out the aqueous reforming of PYA at varying temperatures and over different Lewis-acid catalysts (Figure 2c,d). At neutral pH, increasing the temperature from RT to 70 °C raises LA selectivity from ∼20% to ∼52%, indicating that thermal Cannizzaro steps can operate concurrently with photoredox pathways. Under alkaline conditions, oxygen-vacancy-enriched TiO_2_ (TiO_2_–V_O_) shows enhanced LA selectivity of ∼85% compared to ∼55% over pristine TiO_2_. With sufficient OH^–^ to promote dehydration and/or retro-aldol chemistry, highly selective production of LA can also be achieved using other biomass-derived substrates, including glycerol, fructose, and glucose (Figure 2d and Table S2).^27^ Notably, TiO_2_–V_O_ catalyzed photoreforming of cellulose achieved 41% LA selectivity, suppressing C–C cleavage to C_1–2_ acids that are commonly produced in traditional alkaline processes.^28,29^ These results suggest a photoredox–thermal process that can potentially achieve the PYA-to-LA conversion without high-concentration bases.^30^

Density functional theory (DFT) calculations were conducted to understand the thermal and photoredox pathways in PYA-to-LA conversion over representative facets of rutile TiO_2_ (Figures 3 and S5). On the (110) facet, PYA is first adsorbed via a bidentate bridge of its two oxygen atoms to a Ti center (*E*_ads_ = −0.13 eV). In the Cannizzaro pathway, a nearby adsorbed water molecule dissociates with a lower barrier (Δ*G* ^*‡*^ = 0.55 eV), much lower than O–H bond dissociation in bulk solution (4.77 eV).^31^ The generated *OH then facilitates a nucleophilic attachment at the aldehydic C1 of adsorbed PYA to form an alkoxide intermediate. A subsequent 1,2-hydride shift transfers hydride from C1 to C2, reducing the central carbonyl through a concerted transition state (TS) stabilized by the Ti^4+^ site (Figure 3a). The resulting intermediate has only one coordination bond and therefore easily desorbs from the TiO_2_ surface to yield LA as the final product. Notably, the 1,2-hydride shift is the rate-determining step (RDS) for the Cannizzaro pathway, showing the highest barrier (Δ*G* ^*‡*^ = 1.22 eV), which is consistent with previous thermocatalysis reports.^32^

**Figure 3:**
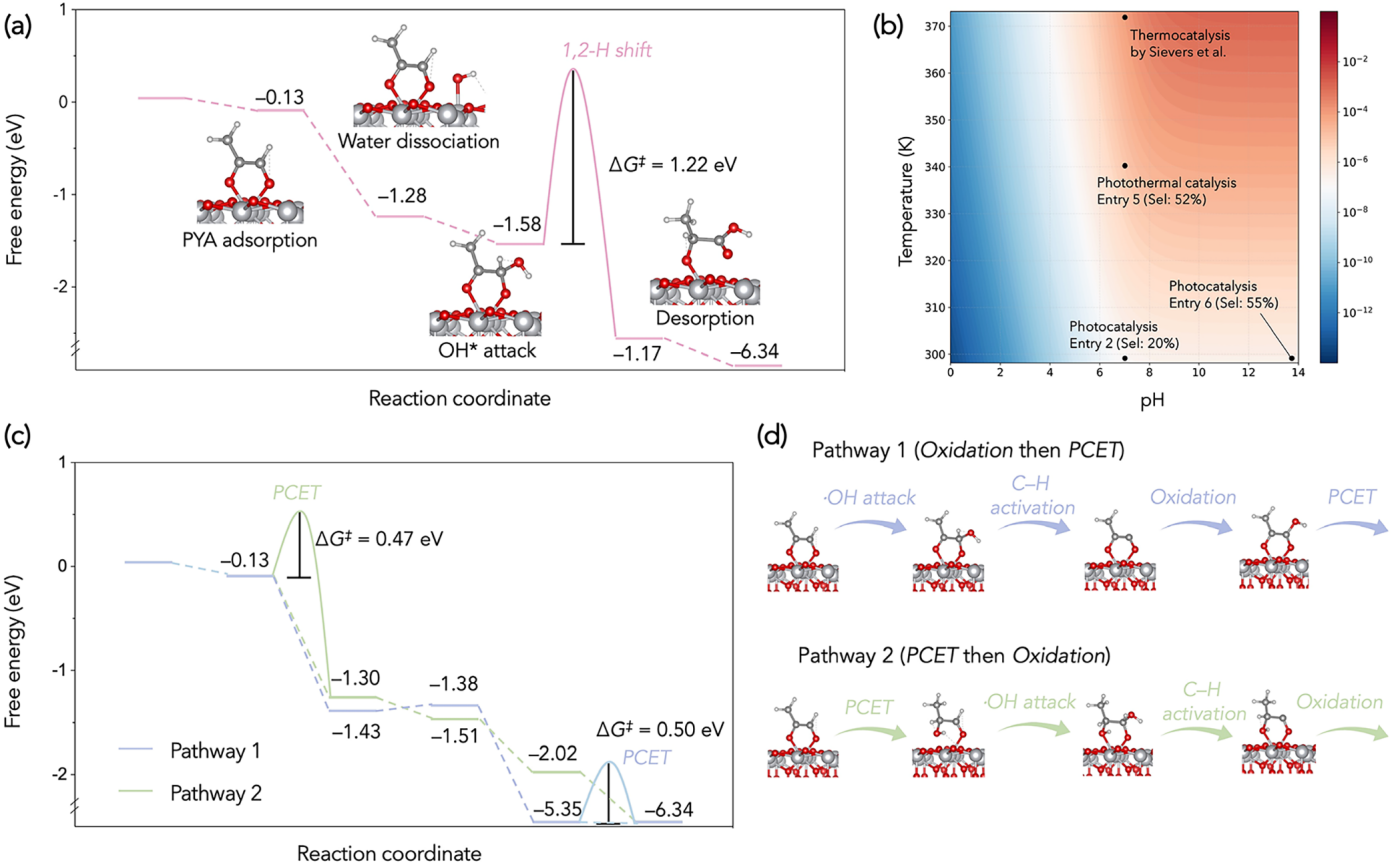
Theoretical calculations of PYA-to-LA conversion over TiO_2_ (110). (a) Energy diagram of the thermal Cannizzaro pathway. (b) Microkinetic analysis showing the relationships between TOF (color legend) and pH/temperature. (c) Energy diagram and (d) intermediate configurations of photoredox pathways.

To further quantify how the high-barrier 1,2-hydride shift reflects on the overall kinetics of PYA-to-LA conversion, we carried out a microkinetic analysis by identifying it as the RDS while assuming quasi-equilibrium or steady-state for other steps (Figure 3b).^33^ With the Arrhenius-type dependence using Δ*G* ^*‡*^ = 1.22 eV, both temperature and OH^–^ concentration (normalized to pH) emerge as critical factors on the overall reaction rate. The calculated turnover frequencies (TOFs) indicate that under neutral pH or at RT (e.g., entries 2 and 6 in Table S2), TOF falls below 10^–6^. This microkinetic analysis also aligns with a thermocatalysis report, where PYA was reformed to LA at a < 60% LA yield (pH = 7 and 140 °C) over TiO_2_.^20^ In contrast, appreciable TOF is only achieved when *T* > 370 K and pH > 10, rationalizing the need for alkaline conditions (e.g. 2 M KOH) to achieve high LA selectivity in Figure 2c and Table S1.

In comparison, the photoredox scheme exhibits faster kinetics but with compromised selectivity due to multiple pathways and unstable intermediates (Scheme 1 and Figure S4).^34^ As depicted in Figure 3c, the disproportionation of PYA can proceed via (1) ^•^OH-mediated oxidation at C2, followed by PCET (Pathway 1) and (2) PCET first, followed by ^•^OH oxidation (Pathway 2). Alternatively, a single PCET step can happen at C1 to give hydroxyacetone (Pathway 3). On both (110) and (001) facets, PCET is the only endothermic step and therefore identified as the RDS across all the pathways, with Δ*G* ^*‡*^ ranging from 0.47 to 0.61 eV (Figures 3c and S5c).^35^ Though the initial ·OH-mediated oxidation in Pathway 1 renders thermodynamically favorable steps before PCET (Figure 3d), Pathways 2 and 3 exhibit lower PCET barriers of 0.47 and 0.24 eV, respectively, so as to generate hydroxyacetone and lactaldehyde as kinetically favored intermediates. This is also suggested by in situ ATR-FTIR. Compared to pyruvic acid, the adsorption of hydroxyacetone and lactaldehyde is much stronger due to two coordination bonds with Ti^4+^ (*E*_ads_ = −0.21 and −0.20 eV), making them more prone to undergo further reactions such as overoxidation. Therefore, the intermediate multiplicity and the over binding of PCET intermediates lead to unsatisfied selectivity of LA under purely photoredox conditions.

Guided by the kinetic discrepancy of Cannizzaro and photoredox pathways, we sought to regulate the photocatalytic pathway in PYA-to-LA conversion by preventing PCET steps and facilitating pyruvic acid as the intermediate. To this end, we engineered plasmonic photothermal catalysts with enhanced Lewis acidity by decorating TiO_2_–V_O_ with Ag or Au nanoparticles, since these photothermal active metals can (1) generate hot electrons that are favorable in oxygen-related activation rather than HER and (2) induce local heat to promote high-barrier chemical reactions by localized surface plasmon resonance (LSPR) effect.^36,37^ As shown in Figures 4a and S6, the photodeposition of 1 wt % metal on rutile TiO_2_–V_O_ rods yields M/TiO_2_–V_O_ with uniformly dispersed nanoparticles of 5–10 nm. X-ray photoelectron spectroscopy (XPS) confirms the corresponding metallic states (Ag^0^: 3d_5/2_, 367.4; 3d_3/2_, 373.4 eV; Au^0^: 4f_7/2_, 83.4 eV, and 4f_5/2_, 87.0 eV; Figures S7 and S8). As irradiated by wide-spectrum light (365–800 nm), Au/TiO_2_–V_O_ delivers the highest LA selectivity of 68% compared to pristine TiO_2_ and Ag/TiO_2_–V_O_ (Table S3). Inductively Coupled Plasma (ICP) analysis suggests the amount of Au loading is around 1.02 wt % (Table S4). X-ray diffraction (XRD) displays the reflections of the rutile TiO_2_ facet and an additional Au (001) facet for Au/TiO_2_–V_O_ (Figure S9). Brunauer–Emmett–Teller (BET) analysis shows the specific area decreases in Au/TiO_2_–V_O_ (20.2 m^2^/g), compared to TiO_2_ (23.2 m^2^/g) and TiO_2_–V_O_ (21.6 m^2^/g) (Figure S10 and Table S5).

**Figure 4:**
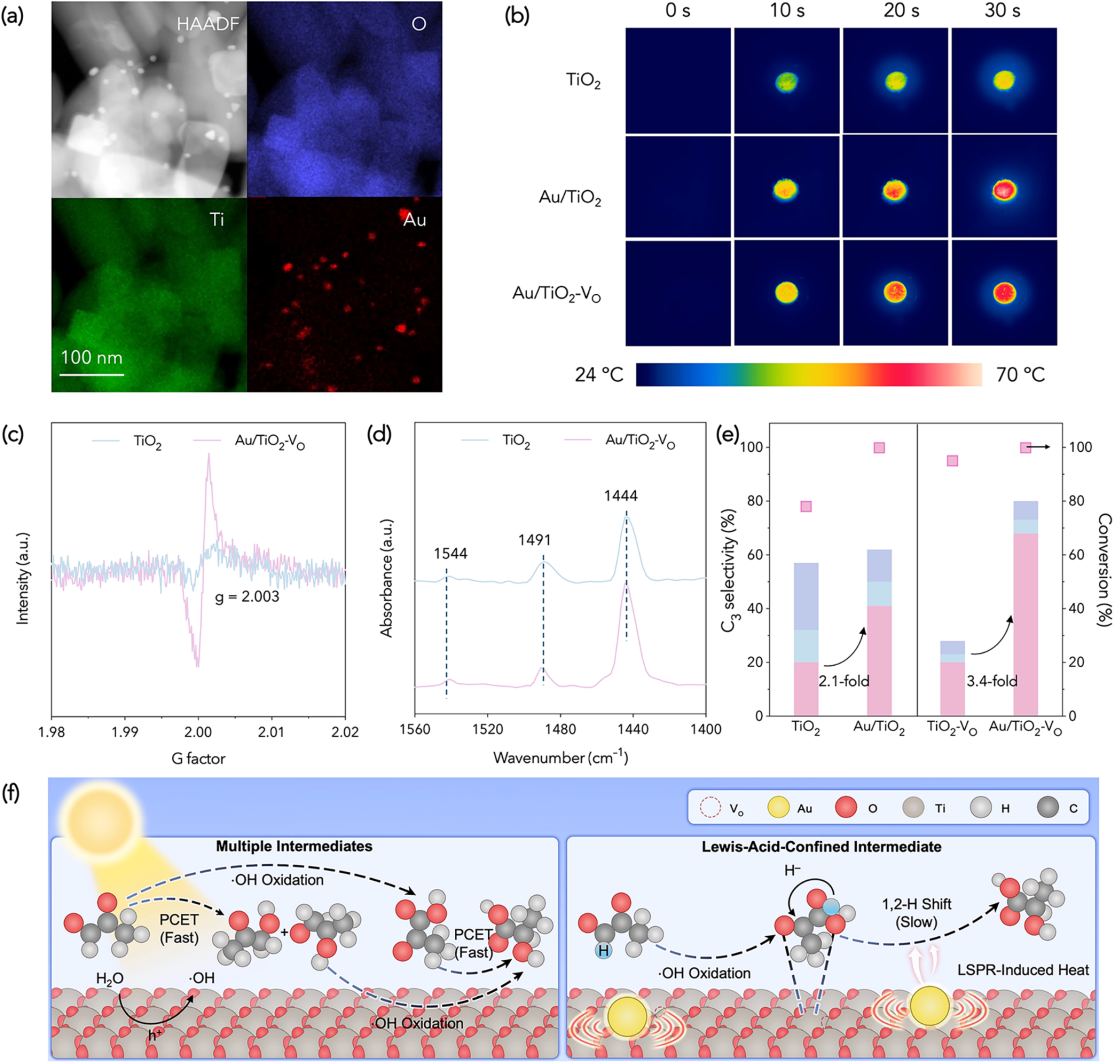
Catalyst characterizations and performance evaluation of Au/TiO_2_–V_O_. (a) HAADF-TEM and elementary mapping, (b) IR monitoring of real-time temperature, (c) low-temperature EPR spectra, (d) py-FTIR spectra, (e) comparison of C_3_ selectivity at neutral pH. (f) Illustrations of solely photoredox on TiO_2_ (left) or thermal Cannizzaro-type reaction on Au/TiO_2_–V_O_ (right).

To confirm the LSPR heat enhanced by both oxygen vacancies and Au loading, we monitored the real-time temperature of the Au/TiO_2_–V_O_ surface by IR imaging (Figure 4b). Upon irradiation, the surface temperature completely rose from 24 to 70 °C within 30 s, higher than those of pristine TiO_2_ (∼45 °C) and Au/TiO_2_ (partially 70 °C). EPR evidence a high density of oxygen vacancies in Au/TiO_2_–V_O_ via the intensified signals at *g* = 2.003 (Figure 4c). Pyridine-adsorption FTIR confirms the presence of both Brønsted (1544 cm^–1^) and Lewis (1444 cm^–1^) acid sites, with the band at 1491 cm^–1^ indicative of contributions from both (Figures 4d and S11).^27^ As shown in Table S6, the quantified acidities show that Au/TiO_2_–V_O_ exhibits remarkably enhanced Lewis acidity (15.94 μmol g^–1^) compared to that of TiO_2_ (8.35 μmol g^–1^), consistent with an increased population of coordinatively unsaturated Ti^4+^ centers generated by oxygen vacancies.

To confirm whether the photothermal catalytic performance varies with the metal sizes, we synthesized Au/TiO_2_–V_O_ of different Au particle sizes. As shown in Figure S12, four catalysts of average diameters (5.8, 8.0, 23.4, and 28.3 nm) exhibit a red shift in the LSPR absorption peak around 550–600 nm. Though the extended adsorption range leads to slightly increased conversions, the highest LA selectivity is achieved when the Au particle size is 8.0 nm, likely due to the trade-off between adsorption efficiency and effective active sites. Notably, the photothermal catalysis over Au/TiO_2_–V_O_ results in much less overoxidized products compared to photocatalysis over TiO_2_ (Table S7), and the reaction kinetic is remarkably enhanced to achieve nearly quantitative conversion of PYA in 2 h (Figure S13). The Arrhenius plotting also suggests the temperature dependency of the Au/TiO_2_–V_O_-based process, with an apparent activation energy of 28.8 kJ mol^–1^ (Figure S14). Dark reactions exhibit completely inhibited and significantly lowered conversions at neutral and 0.1 M KOH, respectively, which suggest the role of light irradiation in the Au/TiO_2_–V_O_-based process (Figure S15). As shown in Figure 4e, at neutral pH, Au/TiO_2_ and Au/TiO_2_–V_O_ deliver 2.1- and 3.4-fold increased LA selectivity compared to TiO_2_ and TiO_2_–V_O_, respectively, demonstrating the promotion effect of LSPR in PYA-to-LA conversion. The use of low-concentration KOH (0.1 M) leads to an unprecedented LA productivity of 130.8 mmol g^–1^ h^–1^.

Moreover, we observed the formation of the carbon-centered intermediate over Au/TiO_2_–V_O_, which is trapped by both quadrupole-time-of-flight (QTOF) mass spectrum and EPR test (Figure S16). The subsequent 1,2-hydride shift as the RDS is further supported by kinetic isotope effect (KIE) testing after the rapid H/D exchange in PYA (Figure S17). These observations support the sequential occurrence of photooxidation and a photothermal 1,2-hydride shift for PYA-to-LA conversion, as shown in Figure 4f. By contrast, ^•^OH and e^–^ species on the TiO_2_ surface drive unregulated photooxidation and PCET steps to yield PYA as well as multiple intermediates, which are prone to desorb or to be further overoxidized. The regulated cascade over Au/TiO_2_–V_O_ leads to a 2.8-fold and 3.4-fold increase in LA selectivity at different pH values (Table S3). Moreover, Au/TiO_2_–V_O_ shows a good reusability and structural durability in 5 recycles (Figure S18), suggesting its promise in large-scale and continuous operation.

## Conclusions

This study proposed a photocatalysis-coupled photothermal Cannizzaro-type route for selective and ambient production of LA from biomass-derived polyols, guided by comparative insights into Cannizzaro and photoredox pathways on TiO_2_. At neutral pH, ^•^OH and e^–^ enabled a cascaded photoredox route for PYA disproportionation, but the selectivity was limited by noncascade parallel pathways yielding weakly adsorbed hydroxyacetone and lactaldehyde, and by overoxidation of strongly adsorbed pyruvic acid. In situ ATR-FTIR and DRIFTS elucidated the kinetically favorable intermediates and temperature dependence of Cannizzaro-type reaction as limited by Lewis-acid coordination and 1,2-hydride shift, respectively. The bifunctional catalyst Au/TiO_2_–V_O_ generated pyruvic acid over PCET intermediates and effectively promoted the 1,2-hydride shift by LSPR-induced heat, which ultimately delivered 3.4- and 2.8-fold increases in LA selectivity compared to TiO_2_ in neutral and 0.1 M KOH solutions, respectively, at room temperature.

## Supplementary Material



## Notes

Experimental details, catalyst characterizations, in situ ATR-FTIR spectra, microkinetic simulations, DFT calculations, and catalytic performance (PDF)
