# Supplementary material for: Selective Biomass Valorization via Cascade Photooxidation and Photothermal Hydride Shift
Source: J Am Chem Soc. 2026 Mar 27;148(15):16232–40. doi: 10.1021/jacs.6c01516 (PMC13107452; doi:10.1021/jacs.6c01516)
Supplement: Supplementary file 1 [file ja6c01516_si_001.pdf]

Supporting Information

Selective Biomass Valorisation via Cascade Photooxidation and Photothermal Hydride Shift

Yingchuan Zhang,[1] Fupeng Zhang,[1] Ruotong Yang,[1] Guangri Jia,[1] Zhuang Ma,[1] Nengchao Luo,[2] Zhengxiao Guo[1]*

[1] Department of Chemistry, The University of Hong Kong, Hong Kong 999077, China

[2] State Key Laboratory of Catalysis, Dalian National Laboratory for Clean Energy, Dalian Institute of Chemical Physics, Chinese Academy of Sciences, Dalian 116023, China

Corresponding author: zxguo@hku.hk

Experimental Procedure	Page 2–9
Figure S1–S18	Page 10–27
Table S1–S7	Page 28–34
References	Page 35–36

Experimental Procedure

Materials

All the chemicals were obtained from commercial sources and used without further purification. Cellulose, glucose, fructose, glycerol, pyruvaldehyde (40 wt% aqueous solution), lactic acid (DL-), hydroxyacetone, lactaldehyde, pyruvic acid, isopropyl alcohol (IPA), solvents, TiO_2 (rutile, 99.995% trace metals basis) and all the metal precursors were purchased from Sigma-Aldrich.

Synthesis of M/TiO_2

M (Pd or Pt)/TiO_2 (1 wt% loading) was synthesized via a photodeposition method. In brief, 100 mg TiO_2 and metal precursors ($H_2PtCl_6 \cdot 6H_2O$/$PdCl_2$) were dispersed in a 10 mL 20% methanol aqueous solution and then irradiated under 365 nm LED with stirring for 3 h. The as-prepared M/TiO_2 was collected by centrifugation, washed with water/ethanol three times and dried at 60 ºC overnight.

Synthesis of TiO_2-V_O

TiO_2-V_O was synthesized by reductive calcination.[1] In brief, the commercial TiO_2 powder was directly calcinated in 5% H_2/Ar at 500 ºC for 3 h to give TiO_2-V_O as a gray powder.

Synthesis of Ag/TiO_2-V_O

Ag/TiO_2-V_O (1 wt% loading) was synthesized via a photodeposition method. In brief, 100 mg TiO_2-V_O with $AgNO_3$ (metal content 1 wt%) was dispersed in 10 mL of 20% methanol aqueous solution, then degassed and stirred for 10 min under irradiation of 365 nm LED. The as-prepared Au/TiO_2-V_O was collected by centrifugation, washed with water/ethanol three times and dried at 60 ºC overnight.

Synthesis of Au/TiO_2-V_O of different particle sizes

Au/TiO_2-V_O (1 wt% loading) was synthesized via a photodeposition method. In brief, 100 mg TiO_2-V_O with $HAuCl_4$ (metal content 1 wt%) was dispersed in 10 mL of 1%, 5%, 20% and 40% methanol aqueous solutions, degassed and stirred for 10 min under irradiation of

365 nm LED. The as-prepared Au/TiO_2-V_O was collected by centrifugation, washed with water/ethanol three times and dried at 60 ºC overnight.

Photocatalytic reactions

Photocatalytic reactions were carried out in a multichannel photoreactor. In detail, to a 25 mL quartz tube were added 5 mg catalyst, 100 μL of PYA solution and 5 mL of DI water. The mixture was degassed and refilled with argon three times, ultrasonicated for 10 min and stirred under the irradiation of 365 nm LED light or 365–800 nm white light (200 mW cm^{-2}) at desired temperatures for 3 h. For photocatalytic entries, the temperature of the reactor was controlled by an external water pump. Photothermal reactions were performed in the same reactor without cooling.

To quantify the products, 1 mL mixture was collected by syringe and filtered through 0.22 μm aqueous membrane. The sample was analysed by high-performance liquid chromatography (HPLC, Vanquish Core, Thermo Fisher Scientific) equipped with a differential refractive index (RI) detector. The compounds were separated by a carbohydrate analysis column (Aminex HPX-87H, Bio-Rad) under a flow (0.6 mL/min) of 5 mM H_2SO_4 solutions. All the compound concentrations were calculated using external standards. The C_3 product selectivity was calculated based on the normalised concentrations of all the detected aqueous products in HPLC. The product yield was calculated by the total carbon numbers of a specific product divided by that of consumed PYA.

In-situ DRIFTS

Diffuse reflaxions infrared Fourier transformations spectroscopy (DRIFTS) was recorded on INVENTIO S (Bruker, Germany) with MCT detector and custom-built cell (Harrick). After degassing, argon containing the PYA solution was continuously pumped (40 mL/min) into the pool where the catalyst was pre-loaded for 30 min to achieve adsorption equilibrium. The signals were recorded each 5 min at desired temperatures under the irradiation of 365 nm light. The resolution is 8 cm^{-1} with a scanning number of 128.

Catalyst characterisations

The transmission electron microscopy (TEM) was recorded on FEI Talos F200X. The photothermal images were recorder on Fotric 226S under the irradiation of an UV spot light at 0.071 W cm^{-2}. Lewis/Brønsted acidity was characterised by pyridine-FTIR on Bruker Tensor 27. The existence of oxygen vacancies (V_O) in the synthesized catalysts was confirmed by low-temperature electron paramagnetic resonance (EPR) at 100 K using Bruker ELEXSYS E500. The generation of hydroxyl radical (·OH) was confirmed on electron paramagnetic resonance (EPR) spectrometer EMSplus-6/1 (Bruker, Germany) using 5,5-dimethyl-1-pyrroline N-oxide (DMPO, Aladdin) as the probe. The mass spectra were collected by LC-MS (Bruker Impact II) with ESI model. In-situ ATR-FTIR was conducted on Nicolet IS50 (Bruker, Germany) equipped with ATR accessory and MCT detector using a pumping method the same with DRIFTS conditions. The relative surface area was calculated on an Autosorb IQ Analyzer (Quantachrome, US) calculated from N_2 uptake isotherm at constant 77 K fitted with Brenauer-Emmet-Teller (BET) method. PXRD was recorded on an X-ray diffractometer (Bruker, Germany) using a Cu-Kα source.

Radical trapping

2,2,6,6-tetramethylpiperidine-1-oxyl (TEMPO, Aladdin) was used to capture carbon-centred radicals. In brief, 5 mg of catalyst, 100 μL of PYA solution in 5 mL of DI water and 0.5 eq. TEMPO were added to the reactor. The mixture was degassed and refilled with argon three times, ultrasonicated for 10 min and stirred under the irradiation of white light for 1 h. The product was subjected to quadrupole-time of mass spectrometer (QTOF, Thermo Fisher Scientific) in a negative ESI mode.

Theoretical calculations

For density functional theory (DFT) calculations, the CP2K software package was used with the generalized gradient approximation functional PBE in combination with the DZVP-MOLOPT-SR-GTH basis set, along with the DFT-D3 dispersion correction scheme.[2–7] The energy cutoff and real-space cutoff were set to be 500 and 50 Ry, respectively. For TiO_2 (rutile), we first performed a cell optimisation of the original structure and then extracted the

(001) and (110) surface to construct slab models, maintaining periodic boundary conditions in the XY directions while adding a 12 Å vacuum layer in the Z-direction to eliminate periodic effects. This model was used for subsequent adsorption energy and chemical reaction calculations. The adsorption energies for reactant, product and intermediate were determined by its adsorption configuration on the TiO2 surface, with the adsorption energy calculated as follow.

$$E_{ads} = E_{AB} - E_A - E_B$$

Furthermore, considering the basis set superposition error (BSSE) effect caused by the 2-zeta basis set, the calculated adsorption energy may be too high. Therefore, the final adsorption energy eliminates the influence of BSSE, then the formular could be as follow.

$$E_{ads} = E_{AB} - E_A - E_B - E_{BSSE}$$

For chemical reactions, single-point energies were obtained through DFT calculations, followed by vibrational analysis to examine the free energy of the system, with the free energy structure provided by the Shermo software package. For the transition state of chemical reactions, we used the CI-NEB method to optimise the transition state structure and identify the rate-determining step of the chemical reaction. In this system, we employed the CHE (computational hydrogen electrode) to investigate the energy of H atom combine with an electron, defined as follow.[8]

$$G(H^+ + e^-) = \frac{1}{2}G(H_2)$$

During the preparation and post-processing stages, the Multiwfn software package was used to generate the initial CP2K input files and process the calculation results[8], while the sobNEB tool was employed to generate the replica coordinates for CI-NEB. For other unspecified parameters, the default settings of the software package were generally used.

During the preparation and post-processing stages, the Multiwfn software package was used to generate the initial CP2K input files and process the calculation results, while the sobNEB tool was employed to generate the replica coordinates for CI-NEB.[9] For other unspecified parameters, the default settings of the software package were generally used. Considering the free energy calculation, the optimized structures were used to performance vibration analysis, then the free energy would be generated as follow,

$$G = E + pV - TS$$

Where the E is the electron energy, p is the pressure, V is the volume and T is the temperature. S indicates the entropy that was calculated from the result of vibration analysis via Shermo package. Other factors that affect thermodynamic quantities, such as zero-point energy and frequency correction factor, are considered. The details of the method were recorded in the user manual and related publications.[10]

Microkinetic analysis

We constructed a microscopic kinetic model to evaluate the heat-driven Cannizzaro reaction pathway. We used θ to represent the surface coverage of different species. Meanwhile, we simplified the steps of the reaction and denoted the reactants and products of the chemical reaction as A-F. Therefore, we obtained the following equations:

Adsorption:

$$A + * \rightarrow A_{ads}$$

$$B + *_{near,A} \rightarrow B_{ads}$$

Reaction:

$$A_{ads} + B_{ads} \xrightarrow{k_1} C_{ads} + *$$

$$C_{ads} \xrightarrow{k_2} D_{ads} + E_{ads}$$

$$D_{ads} + E_{ads} \xrightarrow{k_3} F_{ads} + *$$

Desorption:

$$F_{ads} \rightarrow F + *$$

Then, we have θ_A, θ_B, θ_C, θ_D, θ_E, θ_F to represent the surface coverage of the aforementioned substances, and θ_* represents the coverage of vacancy sites. According to the conservation of coverage, we have the following conditions:

$$\theta_A + \theta_B + \theta_C + \theta_D + \theta_E + \theta_F + \theta_* = 1$$

Then we construct the rate equations for the above steps. For adsorption, we have:

$$r_{des,A} = k_{des,A} \cdot \theta_A$$

Where k represents the rate constant, the subscript "ads" indicates adsorption, "des" indicates desorption, and $[A]$ represents the concentration of A. For species B, which is

OH^-, we have a similar equation:

$$r_{ads,B} = k_{ads,B} \cdot [B] \cdot \theta_* \cdot \theta_A$$

$$r_{des,B} = k_{des,B} \cdot \theta_B$$

The additional multiplier item θ_A here indicates that species B needs to be adsorbed near species A.

For the Reactions happen next to the adsorption, we have the following equations:

$$r_1 = k_1 \cdot \theta_A \cdot \theta_B$$

$$r_3 = k_3 \cdot \theta_D \cdot \theta_E$$

Finally, for the desorption process, we have similar formular:

$$r_{ads,F} = k_{ads,F} \cdot [F] \cdot \theta_*$$

Based on the generation and consumption rates, write the differential equations for the coverage of each species:

$$\frac{d\theta_A}{dt} = k_{des,A} \cdot \theta_A - k_1 \cdot \theta_A \cdot \theta_B$$

$$\frac{d\theta_B}{dt} = k_{ads,B} \cdot [B] \cdot \theta_* \cdot \theta_A - k_{des,B} \cdot \theta_B - k_1 \cdot \theta_A \cdot \theta_B$$

$$\frac{d\theta_C}{dt} = k_1 \cdot \theta_A \cdot \theta_B - k_{-1} \cdot \theta_C \cdot \theta_* - k_2 \cdot \theta_C$$

$$\frac{d\theta_D}{dt} = k_2 \cdot \theta_C - k_{-2} \cdot \theta_D \cdot \theta_E - k_3 \cdot \theta_D \cdot \theta_E$$

$$\frac{d\theta_E}{dt} = k_2 \cdot \theta_C - k_{-2} \cdot \theta_D \cdot \theta_E - k_3 \cdot \theta_D \cdot \theta_E$$

$$\frac{d\theta_F}{dt} = k_3 \cdot \theta_D \cdot \theta_E - k_{des,F} \cdot \theta_F + k_{ads,F} \cdot [F] \cdot \theta_*$$

Since the energy barrier of the second step reaction is the highest, we consider it to be the rate-determining step (RDS). Therefore, we assume that steps 1 and 2 of adsorption, as well as reaction 1, are close to quasi-equilibrium, while reaction 3 and desorption occur more rapidly. This implies that the accumulation of D, E, and F on the surface is relatively low. Based on the aforementioned quasi-equilibrium assumption, we can have the following equation:

$$k_{des,A} \cdot \theta_A$$

$$\theta_A = K_A \cdot [A] \cdot \theta_*$$

$$K_A = \frac{k_{ads,A}}{k_{des,A}}$$

Where K_A actually represents the adsorption equilibrium constant of A. In addition, we also have:

$$k_{ads,B} \cdot [B] \cdot \theta_* \cdot \theta_A = k_{des,B} \cdot \theta_B$$

$$\theta_B = K_B \cdot [B] \cdot \theta_* \cdot \theta_A = K_A \cdot K_B \cdot [A] \cdot [B] \cdot \theta_*^2$$

$$K_B = \frac{k_{ads,B}}{k_{des,B}}$$

For the first step of reaction:

$$k_1 \cdot \theta_A \cdot \theta_B = k_{-1} \cdot \theta_C \cdot \theta_*$$

$$\theta_C = K_1 \cdot \frac{\theta_A \theta_B}{\theta_*}$$

$$K_1 = \frac{k_1}{k_{-1}}$$

Then we have:

$$\theta_C = K_1 \cdot \frac{(K_A \cdot [A] \cdot \theta_*) \cdot (K_A \cdot K_B \cdot [A] \cdot [B] \cdot \theta_*^2)}{\theta_*} = K_1 \cdot K_A^2 \cdot K_B \cdot [A]^2 \cdot [B] \cdot \theta_*^2$$

For the steady states of D and E, we have the following equations:

$$\frac{d\theta_D}{dt} = 0, \frac{d\theta_E}{dt} = 0$$

$$k_2 \cdot \theta_C = k_{-2} \cdot \theta_D \cdot \theta_E + k_3 \cdot \theta_D \cdot \theta_E = k_3' \cdot \theta_D \cdot \theta_E$$

That means $\theta_D \approx \theta_E$, further according to the calculation result, the activation energy barrier for the reverse reaction of the RDS (step2) is extremely high, which means that k_{-2} is very small. Therefore, we can approximately consider that $k_3' \approx k_3$. That means:

$$k_2 \cdot \theta_C = k_3 \cdot \theta_D \cdot \theta_E$$

$$\theta_D \cdot \theta_E = \frac{k_2}{k_3} \cdot \theta_C$$

For product F, we have:

$$\frac{d\theta_F}{dt} = 0$$

$$k_3 \cdot \theta_D \cdot \theta_E = k_{des,F} \cdot \theta_F - k_{ads,F} \cdot [F] \cdot \theta_*$$

Assuming that desorption occurs relatively quickly, then it means $k_{des,F} \gg k_{ads,F} \cdot [F]$, then we have:

$$\theta_F \approx \frac{k_3 \cdot \theta_D \cdot \theta_E}{k_{des,F}}$$

Consider that θ_D, θ_E, θ_F will be small numbers, then we have:

$$\theta_* \approx \frac{1}{1 + K_A \cdot [A] + K_A \cdot K_B \cdot [A] \cdot [B] \cdot \theta_* + K_1 \cdot K_A^2 \cdot [A]^2 \cdot [B] \cdot \theta_*}$$

We assume that θ_* is close to 1, that this value can be omitted in the multiplier term:

$$\theta_* \approx \frac{1}{1 + K_A \cdot [A] + K_A \cdot K_B \cdot [A] \cdot [B] + K_1 \cdot K_A^2 \cdot [A]^2 \cdot [B]}$$

Regarding the overall reaction rate, it was determined by the RDS:

$$r = k_2\theta_C = k_2K_1K_A^2[A]^2[B]\theta_*^2$$

We substitute θ_* into equation above:

$$r = \frac{k_2 \cdot K_1 \cdot K_A^2 \cdot [A]^2 \cdot [B]}{(1 + K_A \cdot [A] + K_A \cdot K_B \cdot [A] \cdot [B] + K_1 \cdot K_A^2 \cdot [A]^2 \cdot [B])^2}$$

Let us simplify the coefficient terms:

$$r = \frac{k \cdot [A]^2 \cdot [B]}{(1 + K_A \cdot [A] + K_A \cdot K_B \cdot [A] \cdot [B] + K_1 \cdot K_A^2 \cdot [A]^2 \cdot [B])^2}$$

$$k = k_2 \cdot K_1 \cdot K_A^2$$

This final result indicates that the equation that occurs on the surface depends on the concentration of reactant A as well as the concentration of B (which is OH^-).

Based on this, we conducted numerical calculations, using temperature and lg$[OH^-]$ concentration as variables, and obtained the corresponding TOF image.

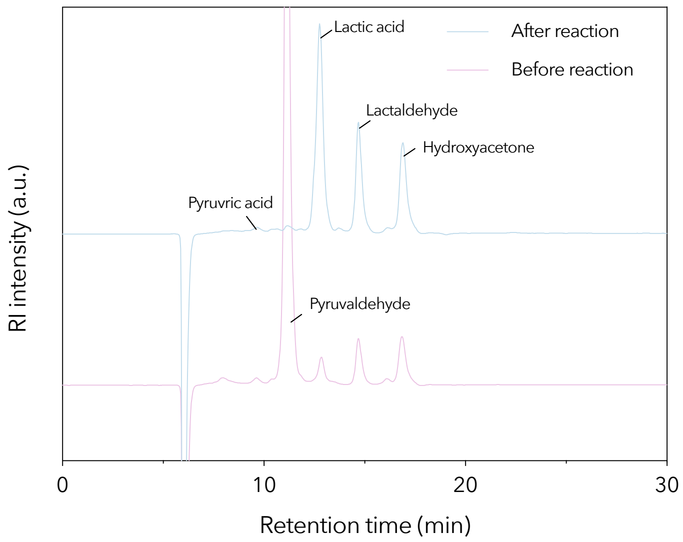

Figure S1. HPLC analysis of reaction mixture.

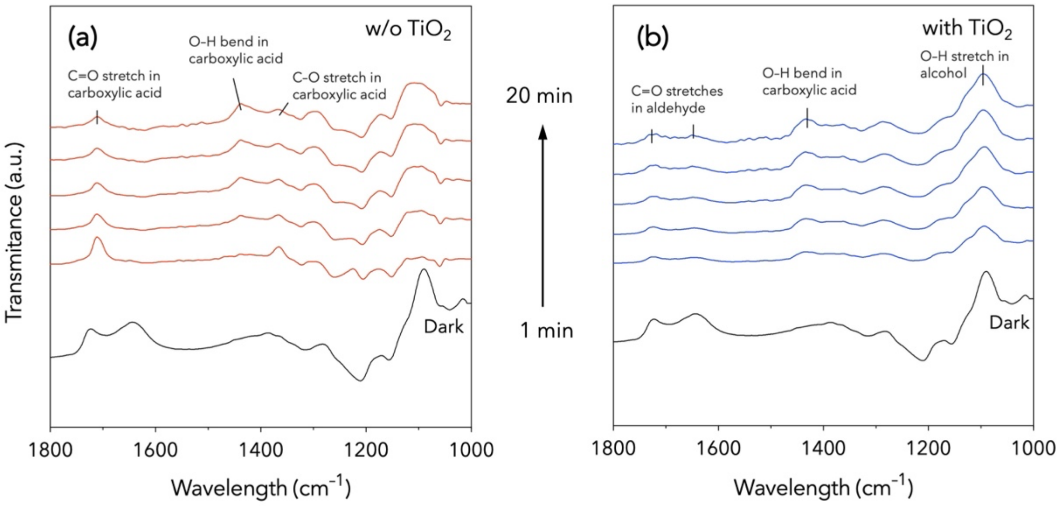

Figure S2. In-situ ATR-FTIR spectra of PYA photoreforming (a) without TiO_2 and (b) with TiO_2. 1707 and 1625 cm^{-1}: C=O stretches in PYA; 1700 cm^{-1}: C=O stretch of –COOH groups in pyruvic acid; 1420 cm^{-1}: O–H bend in pyruvic acid and reduced products; 1320–1200 cm^{-1}: C–O stretches in hydroxyacetone/lactaldehyde/pyruvic acid; 1100 cm^{-1}: O–H stretch of –OH groups in LA.

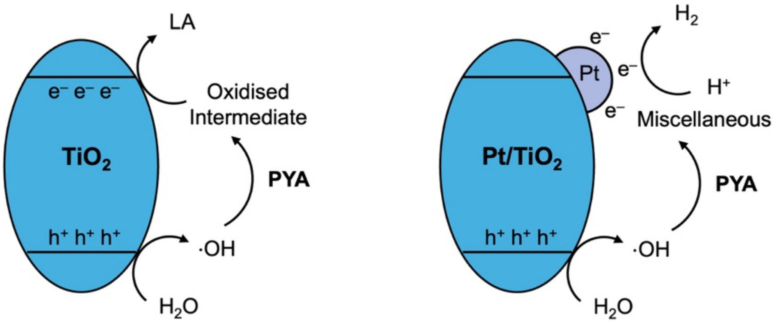

Figure S3. Proposed roles of electrons and holes in PYA reforming over TiO_2 and Pt/TiO_2.

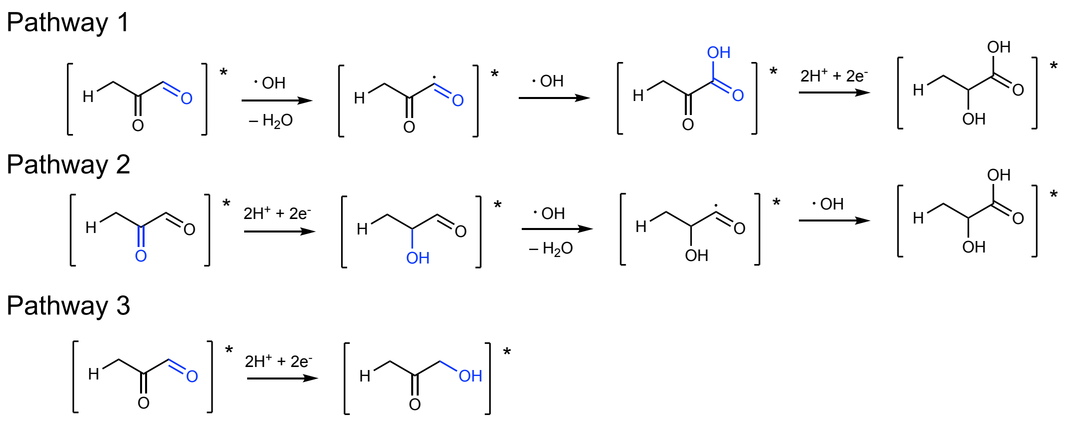

Figure S4. Photocatalytic pathways for 1) PYA-to-LA via pyruvic acid, 2) PYA-to-LA via lactaldehyde and 3) PYA-to-hydroxyacetone.

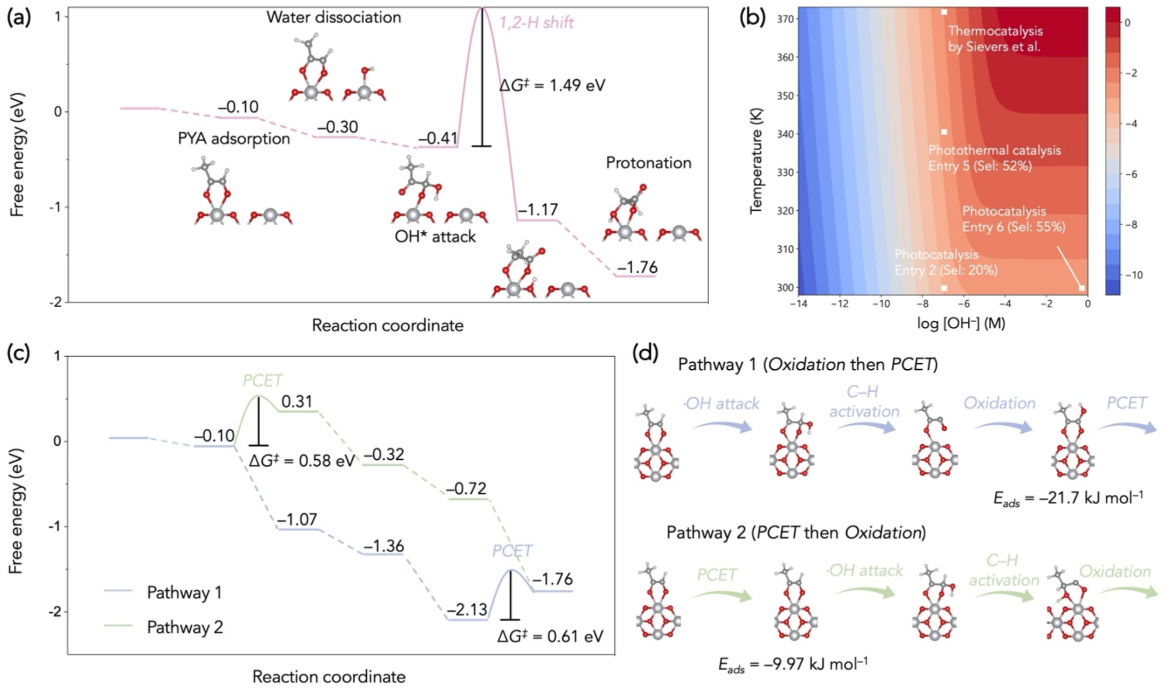

Figure S5. Theoretical calculations of PYA-to-LA conversion over TiO_2 (001). (a) Energy diagram of thermal Cannizzaro pathway. (b) Microkinetic analysis showing the relationships between TOF and pH/temperature. (c) Energy diagram and (d) intermediate configurations of photoredox pathways.

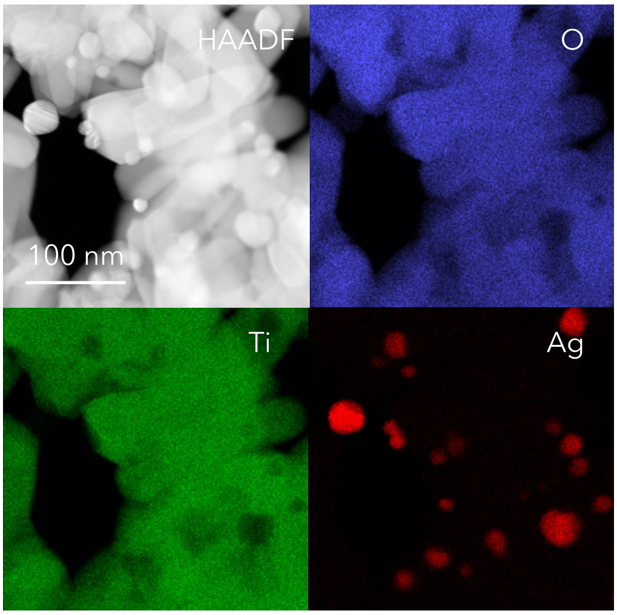

Figure S6. TEM images of Ag/TiO_2-V_O.

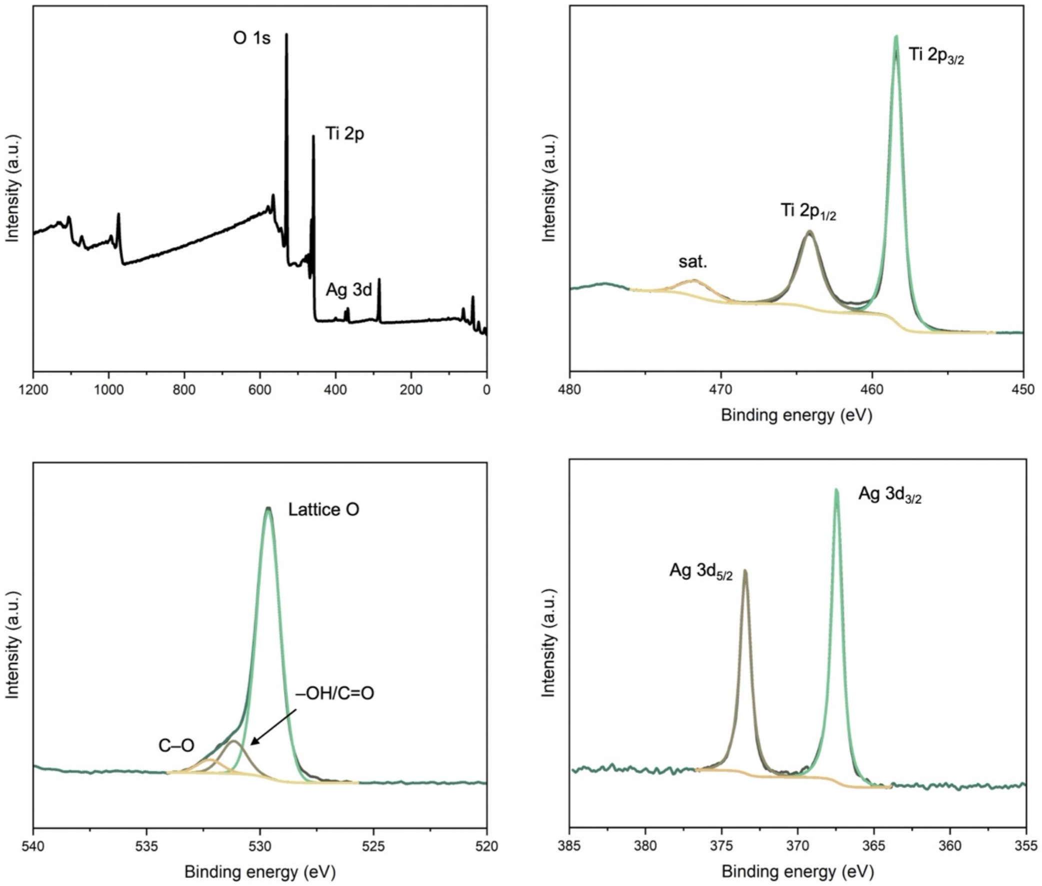

Figure S7. XPS profiles of Ag/TiO_2-V_O.

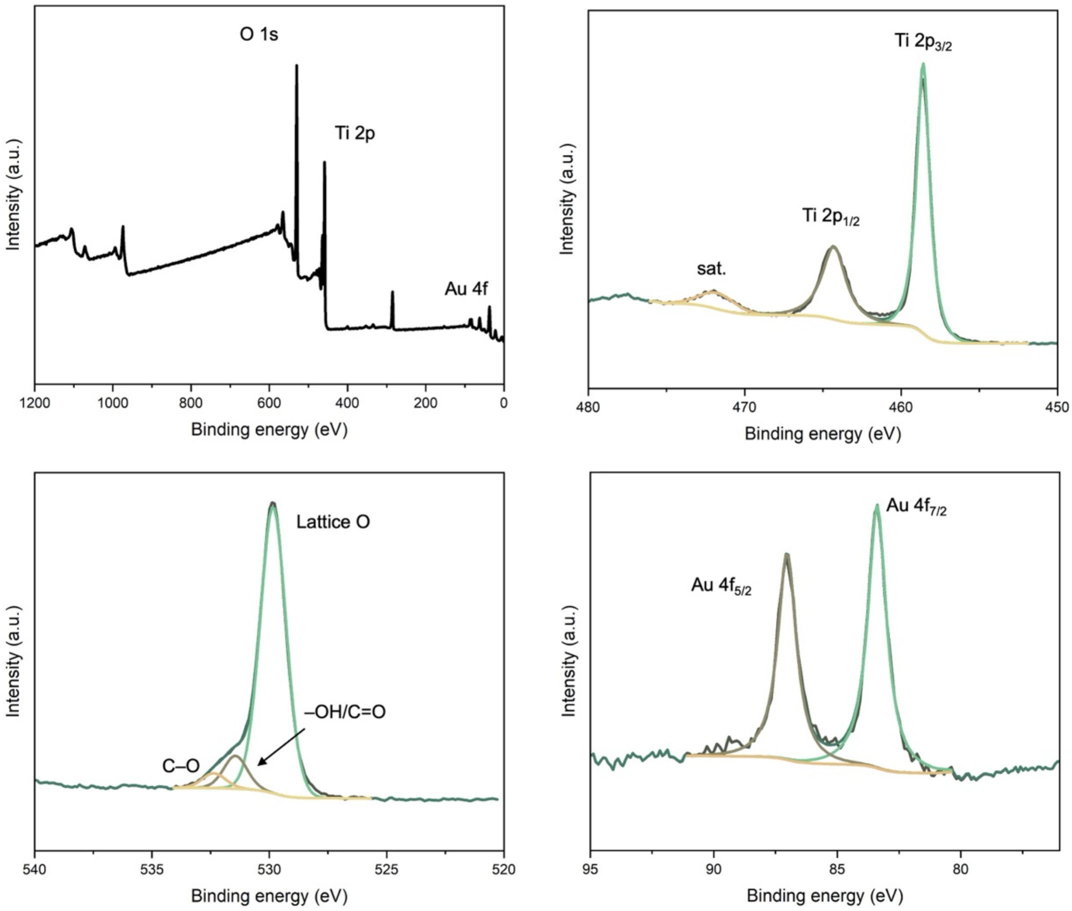

Figure S8. XPS profiles of Au/TiO_2-V_O.

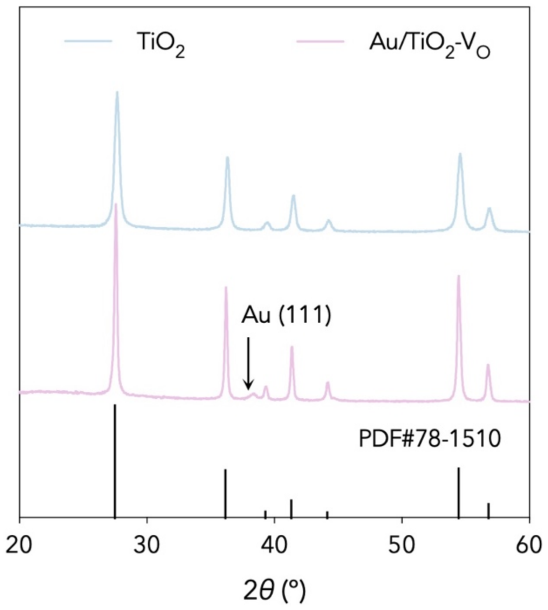

Figure S9. XRD profiles of commercial TiO_2 (rutile) and as-prepared Au/TiO_2-V_O.

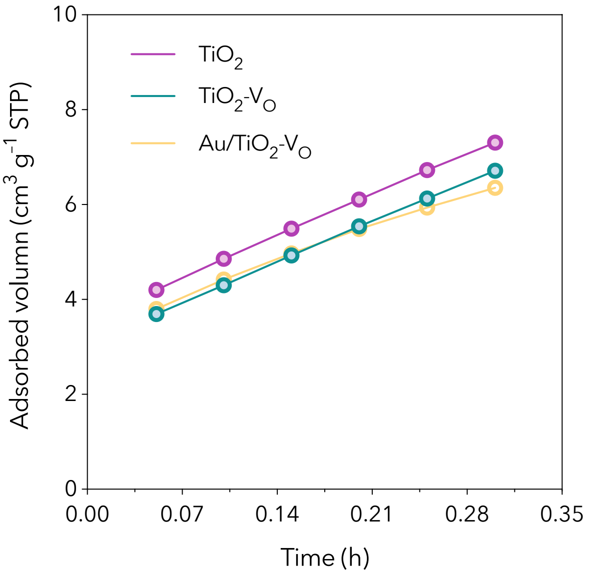

Figure S10. BET curves of different catalysts.

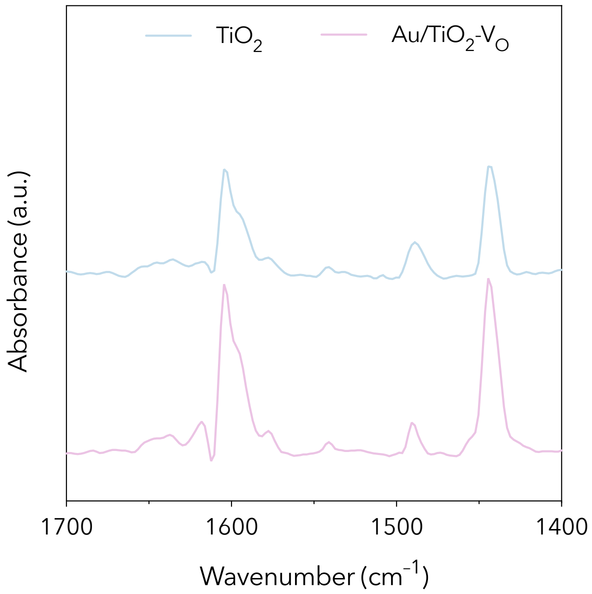

Figure S11. Extended Py-FTIR spectra of different catalysts.

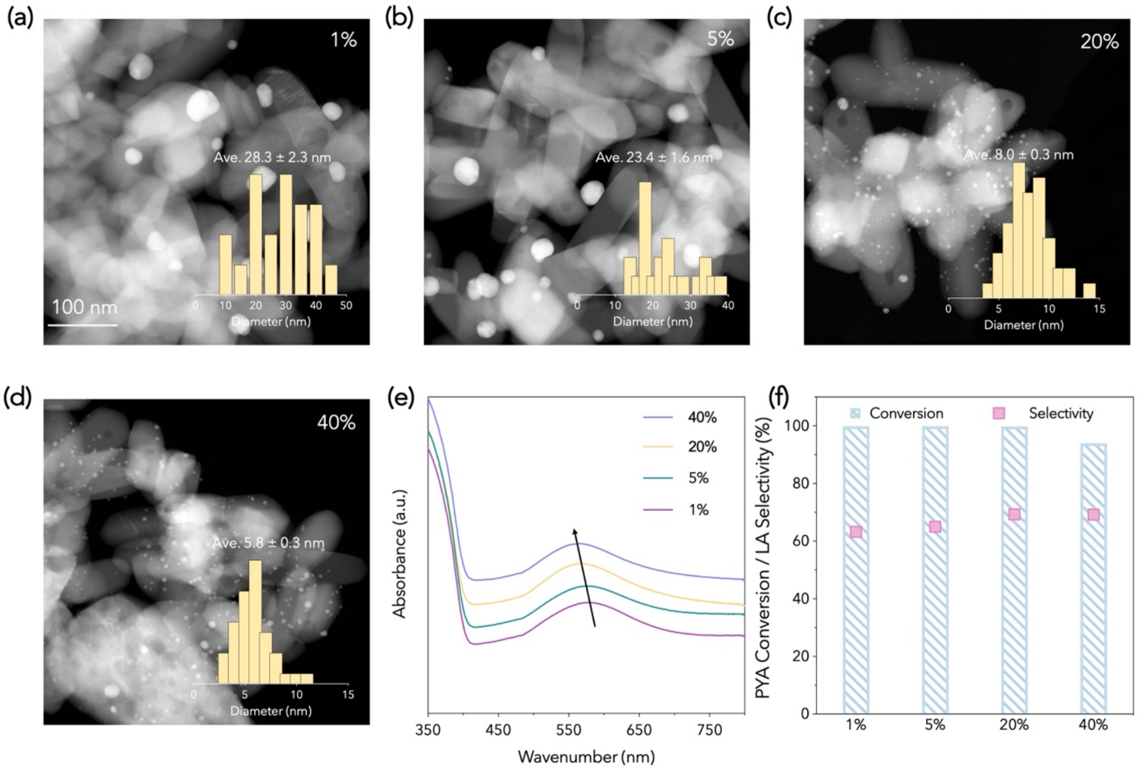

Figure S12. (a–d) HAADF-STEM images and Au particle size distribution. (e) UV-Vis DRS of Au/TiO_2-V_O of different Au particle size. (f) Catalytic performance comparison.

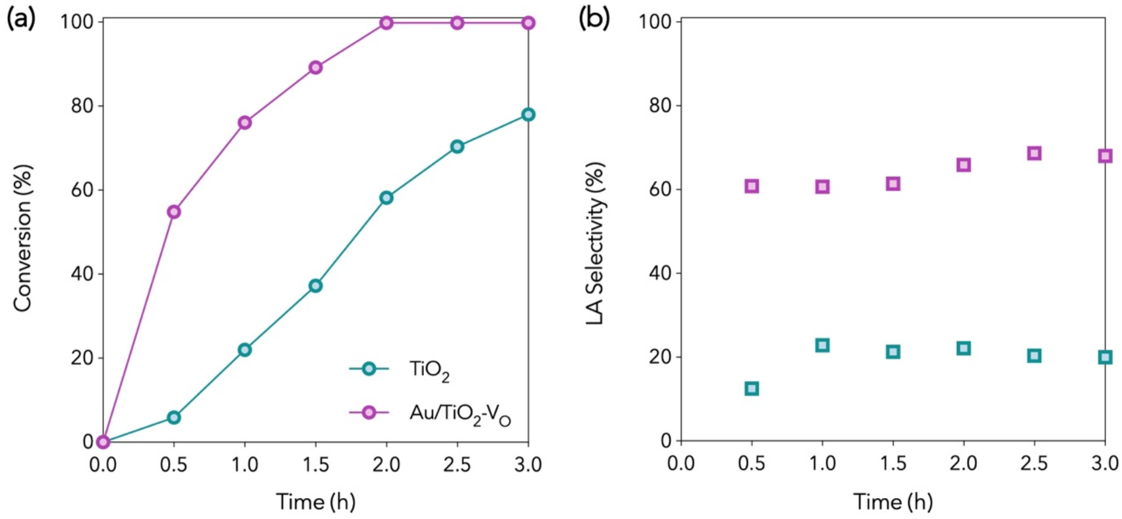

Figure S13. Time-dependent conversions and LA selectivity under photothermal conditions with irradiation of 365 nm for TiO_2 and white light for Au/TiO_2-V_O at neutral pH. (Light intensities: 200 mW cm^{-2})

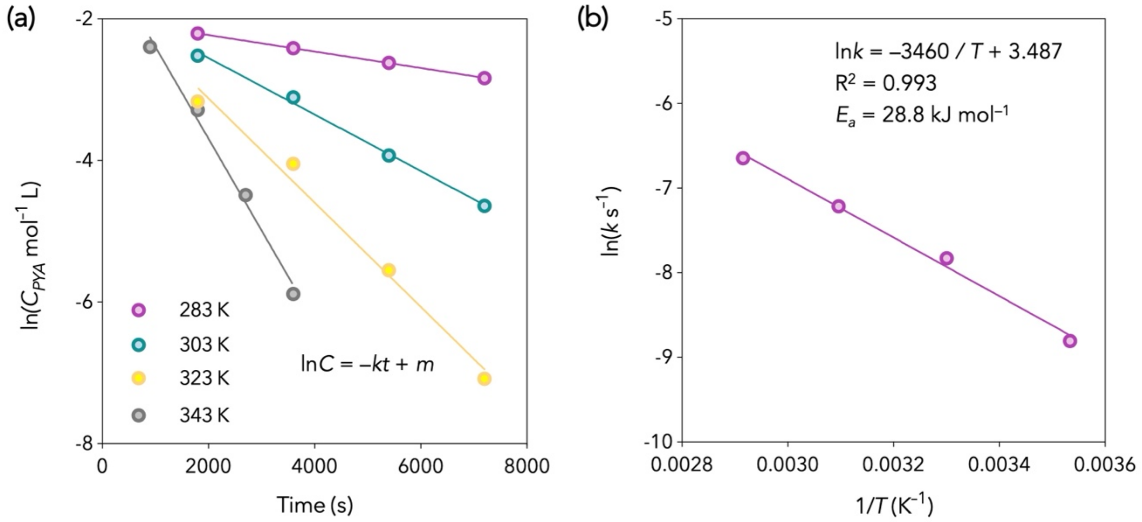

Figure S14. (a) Reaction rate constants *versus* reaction times at different temperatures and (b) Arrhenius plot and calculated apparent activation energy under white light irradiation.

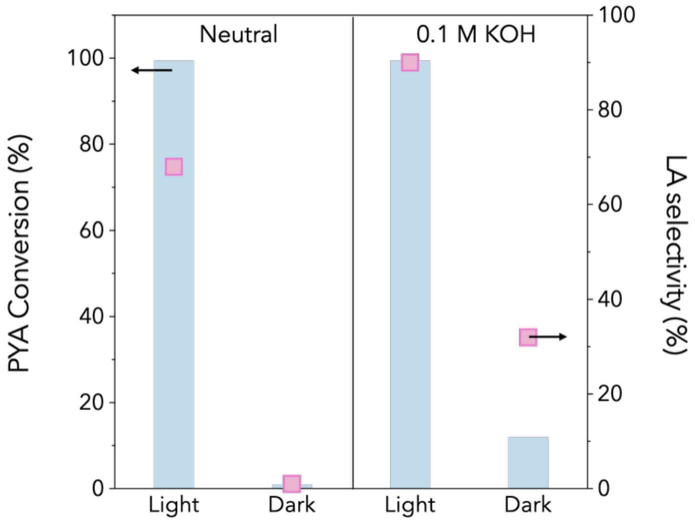

Figure S15. PYA conversions and LA selectivity over Au/TiO_2-V_O with or without irradiation in the first 2 h.

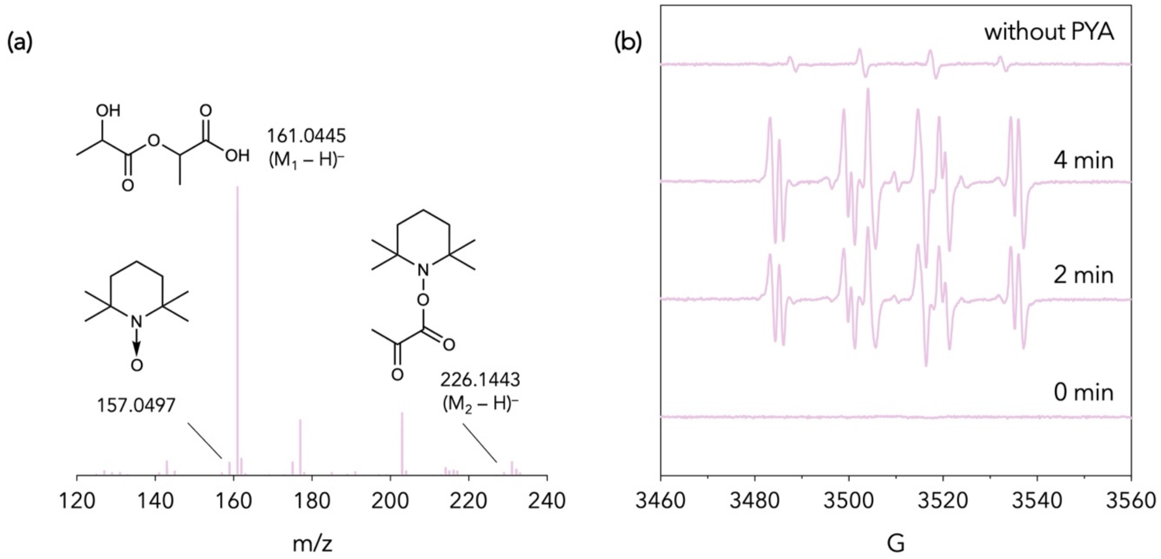

Figure S16. (a) Quadrupole-time of flight (QTOF) mass spectrum of products after adding 2,2,6,6-tetramethylpiperidine1-oxyl (TEMPO) as the radical-trapping reagent (M_1: LA dimer; M_2: Trapped intermediate). (b) EPR spectra of reactions in a PYA solution or pure water using 5,5-Dimethyl-1-pyrroline N-Oxide (DMPO) as the radical-trapping reagent. (The top entry was irradiated in 4 min without PYA to show the signal of ·OH)

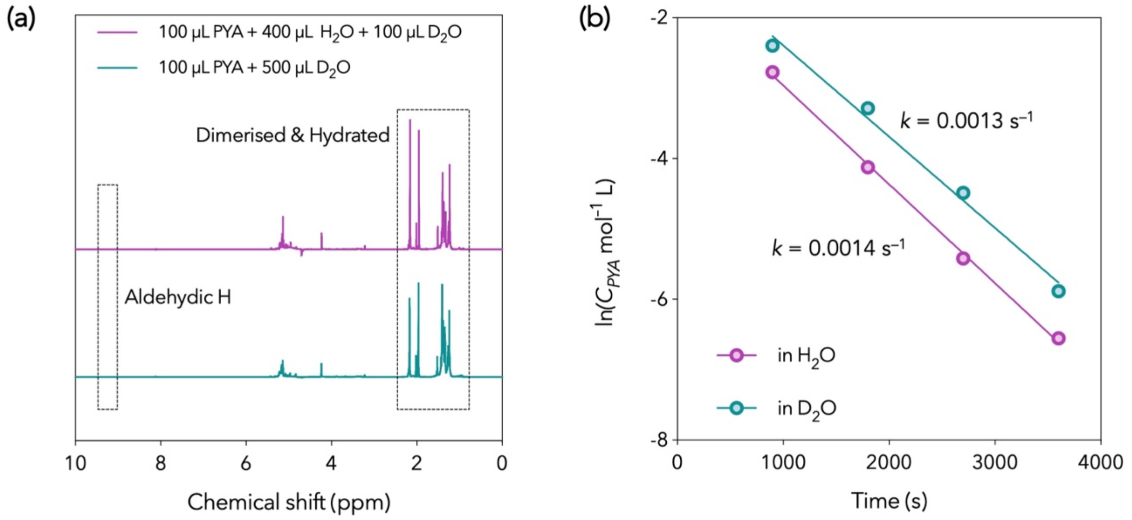

Figure S17. (a) ^{1}H NMR spectra of PYA solutions diluted with H_2O or D_2O. (b) Kinetic constants of standard entries in H_2O or D_2O at 70 ºC.

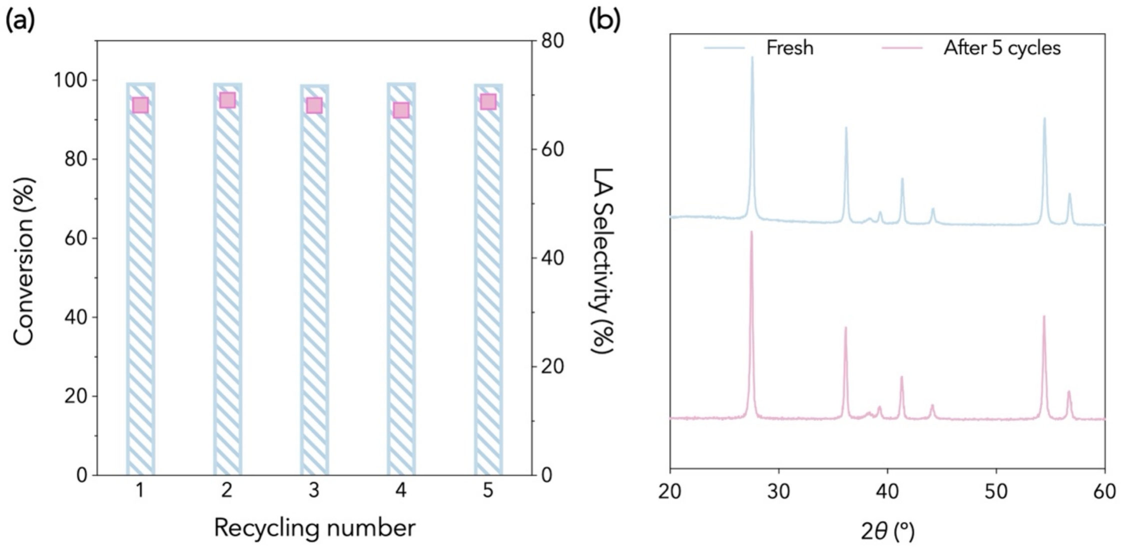

Figure S18. (a) Recycling performance and (b) XRD profiles of Au/TiO_2-V_O before and after 5 recycles.

Table S1. Previous reports for the catalytic conversion of biomass substrates to lactic acid.

Catalyst	Conv. (%)	Yield (%)	Reaction condition	Ref.
**Thermocatalysis for glucose conversion**				
Lead (II) ions	100	71.0	443K, 4 h, 3 MPa, N_2	11
$ErCl_3$	100	76.2	513 K, 0.5 h, 2 MPa, N_2	12
Na_2SiO_3	–	30.0	573 K, 60 s, 8.58 MPa	13
$Ba(OH)_2$	99.5	95.4	298 K, 48 h, 0.1 MPa, N_2	14
Al(III)–Sn(II)	100	81.0	453 K, 2 h, 3.0 MPa, N_2	15
$YNbO_4$-353	96.3	19.6	413 K, 5 h	16
Sn-Beta-NH_2	99.9	56.0	463 K, 2 h	17
γ-Al_2O_3	100	15.0	443 K, 4 h, 1.5 MPa, N_2	18
Sn-Beta	83.3	61.4	473 K, 1 h, 4.0 MPa, N_2	19
**Photocatalysis for glucose conversion**				
B@mCN	100	77.0	333 K, 1.5 h, 2 M KOH, Sunlight	20
$Zn_{1-x}Cd_xS$	90.0	87.0	298 K, 5 h, 1 M NaOH, Xe lamp	21
Ut-OCN	>96.9	63.9	323 K, 1.5 h, 3 M KOH, visible light	22
N-TiO_2	>99.0	84.9	333 K, 2 h, 2 M KOH, solar simulated	23
CuO@CS-H	98.2	54.2	333 K, 1 h, 1.5 M KOH, visible light	24
CNT/LDH	100	88.6	298 K, 2 h, 0.15 M KOH, Xe lamp	25
Pt_x-C_3N_4	100	86.0	303 K, 4 h, 10 M KOH, LED	26

Table S2. Performance evaluation of TiO_2 under different conditions.

Catalyst	Reaction condition[a]	C_3 Selectivity (%)		
		Hydroxyacetone	Lactaldehyde	Lactic acid
None	PYA in neutral, RT, 3 h, Ar	3	2	1
TiO_2	PYA in neutral, RT, 3 h, Ar	12	25	20
Pd-TiO_2	PYA in neutral, RT, 3 h, Ar	5	10	4
Pt-TiO_2	PYA in neutral, RT, 3 h, Ar	4	12	6
TiO_2	PYA in neutral, 70 ºC, 3 h, Ar	3	5	52
TiO_2	PYA in 2M KOH, RT, 3 h, Ar	3	6	55
TiO_2-V_O	PYA in 2M KOH, RT, 3 h, Ar	2	3	85
TiO_2-V_O	Glycerol in 2M KOH, 70 ºC, 3 h, Ar	1	3	64
TiO_2-V_O	Fructose in 2M KOH, 70 ºC, 3 h, Ar	n.d.	n.d.	98
TiO_2-V_O	Glucose in 2M KOH, 70 ºC, 3 h, Ar	3	6	84
TiO_2-V_O	Cellulose in 2M KOH, 70 ºC, 3 h, Ar	2	1	42

n.d.: not detected

The conversions in neutral solutions are among 20–80%, and those in alkaline solutions are nearly 100%.

Table S3. Performance evaluation of metal-doped TiO_2-V_O under different conditions.

Catalyst (pH)	C_3 Selectivity (%)		
	Hydroxyacetone	Lactaldehyde	Lactic acid
TiO_2 (neutral)	12	25	20
Ag/TiO_2-V_O (neutral) [1]	10	7	52
Au/TiO_2-V_O (neutral) [1]	5	7	68
Au/TiO_2-V_O (neutral) [2]	n.d.	n.d.	n.d.
TiO_2 (0.1M KOH)	10	15	34
Ag/TiO_2-V_O (0.1M KOH) [1]	3	5	90
Au/TiO_2-V_O (0.1M KOH) [1]	1	1	95
Au/TiO_2-V_O (0.1M KOH) [2]	1	1	24

[1] White light irradiation

[2] Without light irradiation

The conversions in alkaline solutions are nearly 100%.

Table S4. ICP results of different catalysts.

Catalyst	Ti (wt%)	Au (wt%)
TiO_2	59.71 ± 0.23	–
TiO_2-V_O	60.83 ± 1.00	–
Au/TiO_2-V_O	59.88 ± 0.37	1.02 ± 0.21

Table S5. BET results of different catalysts.

Catalyst	Surface area (m^2/g)
TiO_2	23.3 ± 0.2
TiO_2-V_O	21.6 ± 0.1
Au/TiO_2-V_O	20.2 ± 0.1

Table S6. Acidity properties of different catalysts.

Catalyst	Brønsted (μmol g^{-1})	Lewis (μmol g^{-1})	B/L
TiO_2	0.94	8.35	0.11
Au/TiO_2-V_O	1.45	15.94	0.09

Table S7. Conversion, product yield and carbon balance under photothermal conditions.

	TiO_2 (365 nm)	Au/TiO_2-V_O (White Light)
**Conversion (%)**	78.0	99.9
**Yield (%)**		
Hydroxyacetone	7.1	5.0
Lactaldehyde	14.8	7.0
Lactic acid	11.8	68.3
Pyruvic acid	1.2	1.2
Formic acid	16.2	9.4
CO_2	41.0	4.3
**Carbon balance (%)**	92.1	95.3

Reaction conditions: 100 μL PYA solution, 5 mL DI water, Ar, 3 h, 200 mW cm^{-2} irradiation

References

(1) Li, J.; Zhang, L.; An, X.; Feng, K.; Wang, X.; He, J.; Huang, Y.; Liu, J.; Zhang, L.; Yan, B.; Li, C.; He, L. Tuning Adsorbate-Mediated Strong Metal-Support Interaction by Oxygen Vacancy: A Case Study in Ru/TiO_2. *Angew. Chem. Int. Ed.* **2024**, *63* (31), e202407025.

(2) Kühne, T. D.; Iannuzzi, M.; Del Ben, M.; Rybkin, V. V.; Seewald, P.; Stein, F.; Laino, T.; Khaliullin, R. Z.; Schütt, O.; Schiffmann, F.; Golze, D.; Wilhelm, J.; Chulkov, S.; Bani-Hashemian, M. H.; Weber, V.; Borštnik, U.; Taillefumier, M.; Jakobovits, A. S.; Lazzaro, A.; Pabst, H.; Müller, T.; Schade, R.; Guidon, M.; Andermatt, S.; Holmberg, N.; Schenter, G. K.; Hehn, A.; Bussy, A.; Belleflamme, F.; Tabacchi, G.; Glöß, A.; Lass, M.; Bethune, I.; Mundy, C. J.; Plessl, C.; Watkins, M.; VandeVondele, J.; Krack, M.; Hutter, J. CP2K: An Electronic Structure and Molecular Dynamics Software Package - Quickstep: Efficient and Accurate Electronic Structure Calculations. *J. Chem. Phys.* **2020**, *152* (19), 194103.

(3) Grimme, S.; Antony, J.; Ehrlich, S.; Krieg, H. A Consistent and Accurate *Ab Initio* Parametrization of Density Functional Dispersion Correction (DFT-D) for the 94 Elements H-Pu. *J. Chem. Phys.* **2010**, *132* (15), 154104.

(4) Weigend, F. Accurate Coulomb-Fitting Basis Sets for H to Rn. *Phys. Chem. Chem. Phys.* **2006**, *8* (9), 1057.

(5) Caldeweyher, E.; Bannwarth, C.; Grimme, S. Extension of the D3 Dispersion Coefficient Model. *J. Chem. Phys.* **2017**, *147* (3), 034112.

(6) Wittmann, L.; Gordiy, I.; Friede, M.; Helmich-Paris, B.; Grimme, S.; Hansen, A.; Bursch, M. Extension of the D3 and D4 London Dispersion Corrections to the Full Actinides Series. *Phys. Chem. Chem. Phys.* **2024**, *26* (32), 21379–21394.

(7) Perdew, J. P.; Burke, K.; Ernzerhof, M. Generalized Gradient Approximation Made Simple. *Phys. Rev. Lett.* **1996**, *77* (18), 3865–3868.

(8) Peterson, A. A.; Abild-Pedersen, F.; Studt, F.; Rossmeisl, J.; Nørskov, J. K. How Copper Catalyzes the Electroreduction of Carbon Dioxide into Hydrocarbon Fuels. *Energy Environ. Sci.* **2010**, *3* (9), 1311.

(9) Lu, T. A Comprehensive Electron Wavefunction Analysis Toolbox for Chemists, Multiwfn. *J. Chem. Phys.* **2024**, *161* (8), 082503.

(10) Lu, T.; Chen, Q. Shermo: A General Code for Calculating Molecular Thermochemistry Properties. *Comput. Theor. Chem.* **2021**, *1200*, 113249.

(11) Wang, Y.; Deng, W.; Wang, B.; Zhang, Q.; Wan, X.; Tang, Z.; Wang, Y.; Zhu, C.; Cao, Z.; Wang, G.; Wan, H. Chemical Synthesis of Lactic Acid from Cellulose Catalysed by Lead(II) Ions in Water. *Nat. Commun.* **2013**, *4* (1), 2141.

(12) Lei, X.; Wang, F.-F.; Liu, C.-L.; Yang, R.-Z.; Dong, W.-S. One-Pot Catalytic Conversion of Carbohydrate Biomass to Lactic Acid Using an $ErCl_3$ Catalyst. *Appl. Catal. A - Gen.* **2014**, *482*, 78–83.

(13) Duo, J.; Zhang, Z.; Yao, G.; Huo, Z.; Jin, F. Hydrothermal Conversion of Glucose into Lactic Acid with Sodium Silicate as a Base Catalyst. *Catal. Today* **2016**, *263*, 112–116.

(14) Li, L.; Shen, F.; Smith, R. L.; Qi, X. Quantitative Chemocatalytic Production of Lactic Acid from Glucose under Anaerobic Conditions at Room Temperature. *Green*

Chem. **2017**, *19* (1), 76–81.

(15) Deng, W.; Wang, P.; Wang, B.; Wang, Y.; Yan, L.; Li, Y.; Zhang, Q.; Cao, Z.; Wang, Y. Transformation of Cellulose and Related Carbohydrates into Lactic Acid with Bifunctional Al(III)–Sn(II) Catalysts. *Green Chem.* **2018**, *20* (3), 735–744.

(16) Kim, M.; Ronchetti, S.; Onida, B.; Ichikuni, N.; Fukuoka, A.; Kato, H.; Nakajima, K. Lewis Acid and Base Catalysis of $YNbO_4$ Toward Aqueous-Phase Conversion of Hexose and Triose Sugars to Lactic Acid in Water. *ChemCatChem* **2020**, *12* (1), 350–359.

(17) Shen, Z.; Kong, L.; Zhang, W.; Gu, M.; Xia, M.; Zhou, X.; Zhang, Y. Surface Amino-Functionalization of Sn-Beta Zeolite Catalyst for Lactic Acid Production from Glucose. *RSC Adv.* **2019**, *9* (33), 18989–18995.

(18) Kiatphuengporn, S.; Junkaew, A.; Luadthong, C.; Thongratkaew, S.; Yimsukanan, C.; Songtawee, S.; Butburee, T.; Khemthong, P.; Namuangruk, S.; Kunaseth, M.; Faungnawakij, K. Roles of Acidic Sites in Alumina Catalysts for Efficient D -Xylose Conversion to Lactic Acid. *Green Chem.* **2020**, *22* (24), 8572–8583.

(19) Zhang, Y.; Luo, H.; Kong, L.; Zhao, X.; Miao, G.; Zhu, L.; Li, S.; Sun, Y. Highly Efficient Production of Lactic Acid from Xylose Using Sn-Beta Catalysts. *Green Chem.* **2020**, *22* (21), 7333–7336.

(20) Ma, J.; Li, Y.; Jin, D.; Ali, Z.; Jiao, G.; Zhang, J.; Wang, S.; Sun, R. Functional B@ *m* CN-Assisted Photocatalytic Oxidation of Biomass-Derived Pentoses and Hexoses to Lactic Acid. *Green Chem.* **2020**, *22* (19), 6384–6392.

(21) Zhao, H.; Li, C.-F.; Yong, X.; Kumar, P.; Palma, B.; Hu, Z.-Y.; Van Tendeloo, G.; Siahrostami, S.; Larter, S.; Zheng, D.; Wang, S.; Chen, Z.; Kibria, M. G.; Hu, J. Coproduction of Hydrogen and Lactic Acid from Glucose Photocatalysis on Band-Engineered $Zn_{1-x}Cd_xS$ Homojunction. *iScience* **2021**, *24* (2), 102109. h

(22) Ma, J.; Li, Y.; Jin, D.; Yang, X.; Jiao, G.; Liu, K.; Sun, S.; Zhou, J.; Sun, R. Reasonable Regulation of Carbon/Nitride Ratio in Carbon Nitride for Efficient Photocatalytic Reforming of Biomass-Derived Feedstocks to Lactic Acid. *Appl. Catal. B - Environ.* **2021**, *299*, 120698.

(23) Cao, Y.; Chen, D.; Meng, Y.; Saravanamurugan, S.; Li, H. Visible-Light-Driven Prompt and Quantitative Production of Lactic Acid from Biomass Sugars over a N-TiO_2 Photothermal Catalyst. *Green Chem.* **2021**, *23* (24), 10039–10049.

(24) Li, Y.; Ma, J.; Jin, D.; Jiao, G.; Yang, X.; Liu, K.; Zhou, J.; Sun, R. Copper Oxide Functionalized Chitosan Hybrid Hydrogels for Highly Efficient Photocatalytic-Reforming of Biomass-Based Monosaccharides to Lactic Acid. *Appl. Catal. B - Environ.* **2021**, *291*, 120123.

(25) Ye, X.; Shi, X.; Zhong, H.; Wang, T.; Duo, J.; Jin, B.; Jin, F. Photothermal Strategy for the Highly Efficient Conversion of Glucose into Lactic Acid at Low Temperatures over a Hybrid Multifunctional Multi-Walled Carbon Nanotube/Layered Double Hydroxide Catalyst. *Green Chem.* **2022**, *24* (2), 813–822.

(26) Wang, E.; Mahmood, A.; Chen, S.-G.; Sun, W.; Muhmood, T.; Yang, X.; Chen, Z. Solar-Driven Photocatalytic Reforming of Lignocellulose into H_2 and Value-Added Biochemicals. *ACS Catal.* **2022**, *12* (18), 11206–11215.
